# Qualification of NISTmAb charge heterogeneity control assays

**DOI:** 10.1007/s00216-017-0816-6

**Published:** 2018-02-09

**Authors:** Abigail Turner, John E. Schiel

**Affiliations:** 1 National Institute of Standards and Technology, Institute for Bioscience and Biotechnology Research, 9600 Gudelsky Dr, Rockville, MD 20850 USA; 2 Present Address: Medimmune, LLC, 55 Watkins Mill Rd, Gaithersburg, MD 20878 USA

**Keywords:** Reference Material, NISTmAb, Monoclonal antibody, Biotherapeutic, Biopharmaceutical, System suitability, Biosimilar, Capillary electrophoresis

## Abstract

The NISTmAb is a monoclonal antibody Reference Material from the National Institute of Standards and Technology; it is a class-representative IgG1κ intended serve as a pre-competitive platform for harmonization and technology development in the biopharmaceutical industry. The publication series of which this paper is a part describes NIST’s overall control strategy to ensure NISTmAb quality and availability over its lifecycle. In this paper, the development and qualification of methods for monitoring NISTmAb charge heterogeneity are described. Capillary zone electrophoresis (CZE) and capillary isoelectric focusing (CIEF) assays were optimized and evaluated as candidate assays for NISTmAb quality control. CIEF was found to be suitable as a structural characterization assay yielding information on the apparent pI of the NISTmAb. CZE was found to be better suited for routine monitoring of NISTmAb charge heterogeneity and was qualified for this purpose. This paper is intended to provide relevant details of NIST’s charge heterogeneity control strategy to facilitate implementation of the NISTmAb as a test molecule in the end user’s laboratory.

**Electronic supplementary material:**

The online version of this article (10.1007/s00216-017-0816-6) contains supplementary material, which is available to authorized users.

**Graphical Abstract:**
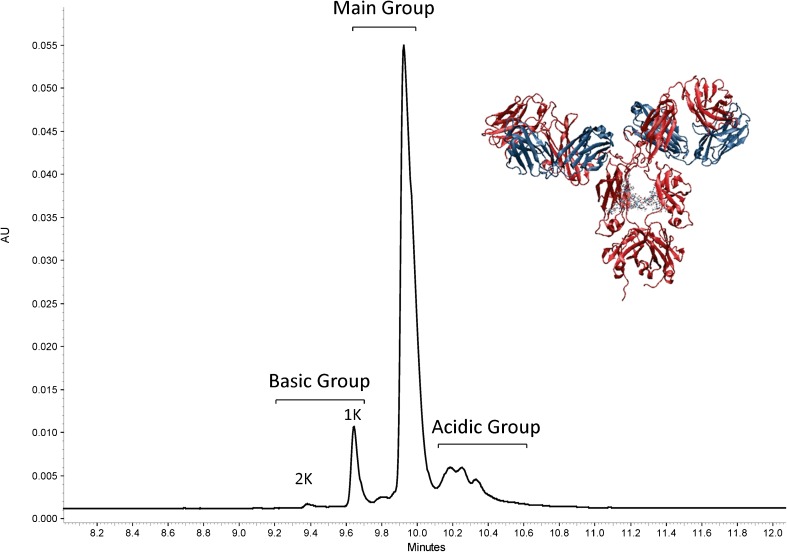
Representative capillary zone electropherogram of the NIST monoclonal antibody (NISTmAb). The NISTmAb is a publicly available research tool intended to facilitate advancement of biopharmaceutical analytics.

## Introduction

Complex biotherapeutics, in particular monoclonal antibodies, increasingly dominate the arena of new drugs submitted for regulatory approval. Quality control mechanisms and their accompanying analytics, originally designed to support small molecule drug development, are still evolving to meet the increasingly sophisticated needs of the biotherapeutics market. Biotherapeutic production in biological systems results in inherently heterogeneous drug products which cannot be fully characterized by any one analytical method, necessitating an analytical toolkit comprised of multiple orthogonal methods [1–3]. Selection of the appropriate testing strategy is guided by the attribute-specific information each method can provide regarding product quality. Increased knowledge surrounding fundamental measurement principles of each measurement can lead to better, more streamlined product characterization and will facilitate new technology and product development. With this need in mind, NIST developed Reference Material 8671 (“NISTmAb”), a class-representative humanized IgG1κ monoclonal antibody which has undergone extensive analytical and biophysical characterization by NIST and industry/academic/government stakeholders [4–15]. The NISTmAb is intended to serve as a platform for open innovation and technology development through evaluation of established and novel analytical methods. In support of this goal, NIST has an ongoing characterization and quality monitoring program using state-of-the-art analytical methods to contribute to a growing open-source knowledge base surrounding the NISTmAb. Here, we describe the development and qualification of charge heterogeneity monitoring assays based on capillary electrophoresis, which form an integral part of the quality control strategy of the NISTmAb Reference Material. This paper is part of a series of articles in this issue describing all aspects of the NISTmAb quality control strategy.

Charge heterogeneity of monoclonal antibodies may reflect a number of post-translational modifications and structural variants which affect product efficacy to varying degrees. Routine monitoring of mAb charge heterogeneity is an integral part of any product quality and lifecycle management plan. Charge sensitive analytical methods commonly employed for mAb charge heterogeneity characterization and monitoring include capillary isoelectric focusing (CIEF) [16, 17], imaged capillary isoelectric focusing (ICIEF) [18], capillary zone electrophoresis (CZE) [19–21], ion exchange chromatography (IEX) [22–24], and free-flow electrophoresis (FFE) [25]. Capillary and microchip-based techniques [26] afford very high separation efficiency even for very rapid separations (<15 min), while consuming very little sample. However, fractionation and sample collection is impractical on these platforms. Preparative scale methods such as OFFGEL, Rotofor, FFE and IEX offer alternative charge variant separations that are amenable to fraction collection and downstream characterization.

In this paper, we present two optimized charge-sensitive assays for characterization and quality control of NISTmAb charge heterogeneity. The NISTmAb in-house Primary Sample (PS) 8670 was utilized to optimize and evaluate charge-based electrophoretic assays and establish their utility in the material’s lifecycle management. We have focused on capillary electrophoresis-based assays for routine monitoring of charge heterogeneity; orthogonal methods including IEX have been developed elsewhere [11]. NISTmAb variants contributing to charge heterogeneity have been identified by mass spectrometry [12] and include multiple asparagine deamidations, C-terminal lysine variants, N-terminal pyroglutamate, lysine glycation, and sialic acid-containing glycoforms. An optimized mobilized CIEF assay is presented which is intended as a characterization test for NISTmAb charge variant apparent isoelectric point (pI), while a rapid and sensitive CZE assay is presented which has been qualified for monitoring of NISTmAb charge heterogeneity.

## Materials and methods

### Materials

The NISTmAb Primary Sample 8670 (PS 8670) is derived from a single production lot and used as the in-house primary sample [27]. PS 8670 is formulated in 12.5 mmol/L L-histidine/12.5 mmol/L L-histidine HCl, pH 6.0 (formulation buffer) at 10 mg/mL.The Advanced cIEF Starter Kit (PN A80976) containing cIEF gel buffer, pI marker peptides, and neutral coated capillary (50 μm internal diameter) was purchased from Sciex Separations. This kit contains four pI markers (4.1, 5.5, 7.0, 9.5, and 10) which are also available from Sciex as a separate pI Marker Kit (PN A58481). An additional pI 8.7 marker (PN 89357-200UL) was purchased from Sigma Aldrich. Bare fused silica capillaries (50 μm inner diameter, 67 cm total length) were purchased from Sciex Separations (PN 338451) and cut to 30.5 or 50.5 cm prior to use. Acidic wash solution (0.1 mol/L hydrochloric acid, in SDS-MW kit PN 390953) and the CZE IQ standard peptide (WYKK, pI 10.0, in pI Marker kit PN A58481) were purchased from Sciex Separations. Iminodiacetic acid (PN 220000-25G), sodium hydroxide (PN 35255-1 L-R), glacial acetic acid (PN A6283-500ML), phosphoric acid (PN 79617-250ML), arginine (PN A5006-100G), ε-aminocaproic acid (EACA, PN A7824-25G), triethylenetetraamine (TETA, PN 90460-10ML), sodium phosphate dibasic dihydrate (PN 71633-250G), and hydroxypropyl-methylcellulose (HPMC, PN H7509-100G) were purchased from Sigma Aldrich. Urea (PN 1084870500) and PVDF syringe filters (PN SLSV025LS) were purchased from EMD Millipore. The broad range (BR) Pharmalyte 3–10 (PN 17–0456-01) and narrow range (NR) Pharmalyte 8–10.5 (PN 17–0455-01) were purchased from GE Life Science. Anhydrous sodium acetate was from Fluka (PN 71183). Polysorbate-20 (Tween™ 20, 10% solution under inert gas, PN 28320) ampules and Zeba columns used for buffer exchange (PN 89882) were purchased from Life Technologies. L-histidine monohydrochloride (PN 2081–06) and L-histidine (PN 2080–05) were from J.T. Baker.

### CIEF solutions and sample preparation

CIEF solutions were as follows: anolyte, 200 mmol/L phosphoric acid; catholyte, 300 mmol/L sodium hydroxide; chemical mobilizer, 350 mmol/L acetic acid; anodic stabilizer, 200 mmol/L iminodiacetic acid; cathodic stabilizer, 500 mmol/L arginine; capillary cleaning solution, 4.3 mol/L urea; urea-gel for separation, 1.5 mol/L (or as indicated) urea in CIEF gel buffer. CIEF solutions were prepared according to the instructions in the manufacturer’s method [28]. Samples at the target concentration (0.4 mg/mL) were prepared by diluting 10 μL of primary sample (PS) 8670 (10 mg/mL) with 240 μL of master mix. The master mix was prepared according to Table 1. The urea concentration and ampholyte composition were optimized as discussed below in the main text. The optimal conditions were determined to be 1.5 mol/L urea and an ampholyte composition of 25% Pharmalyte 3–10:75% Pharmalyte 8–10.5 (1:3 BR:NR) The optimized method utilized the pI 8.7, 9.5, and 10.0 markers as discussed below in the main text. The master mix was vortexed 3 times for 30 s each to ensure complete homogenization. Samples diluted in master mix were vortexed for 30 s each and briefly centrifuged before transfer to CE vials for analysis.

**Table 1:** CIEF Master Mix Components

Reagent	Volume (per sample; for n samples, multiply by n + 1)
Urea in CIEF gel buffer	200 μL
Cathodic Stabilizer	20 μL
Anodic Stabilizer	2 μL
Ampholyte	12 μL
pI Markers	2 μL each

Optimal conditions: 1.5 mol/L urea in CIEF gel buffer; cathodic stabilizer, 500 mmol/L arginine; anodic stabilizer, 200 mmol/L iminodiacetic acid; ampholyte, 25% Pharmalyte 3–10:75% Pharmalyte 8–10.5 (1:3 BR:NR); pI 8.7, 9.5, and 10.0 markers

### CIEF instrumental method

CIEF analyses were performed on a Sciex Separations PA800 plus Pharmaceutical Analysis system fitted with a neutral coated CIEF capillary (Sciex PN 477441), 50 μm i.d., cut to 30.5 cm (20 cm to detector). The system was configured with the UV detector module for absorbance detection at 280 nm. The standard instrumental methods for CIEF designed by the instrument manufacturer were used (see Electronic Supplementary Material (ESM) Tables S8, S9, S10, S11, and S12). Electropherograms were analyzed using the 32Karat software package (Sciex Separations) as described in the CIEF data analysis section of the ESM.

### CZE solutions and sample preparation

Primary Sample 8670 (10 mg/mL) was thawed from −80 °C to room temperature, inverted 3× to 5× to homogenize vial contents, then either portioned into aliquots and stored at −80 °C or maintained at 2 °C to 8 °C up to 1 month. Prior to analysis, Primary Sample 8670 (10 mg/mL) was diluted to the indicated concentration with type 1 deionized ultrafiltered (DIUF) water and mixed. The target loading concentration for the assay was 1.5 mg/mL. The optimized background electrolyte (BGE) employed for CZE analysis contained 400 mmol/L 6-aminocaproic acid (EACA), 2 mmol/L triethylenetetraamine (TETA), acetic acid (pH 5.7), and 0.03% (*w*/*v*) Tween™ 20. The detailed preparation protocol for this and other BGE tested during optimization are listed in the CZE ESM section.

### CZE instrumental method

All samples were analyzed using a Sciex Separations PA800 *plus* pharmaceutical analysis system. Samples were detected at the capillary window, 40 cm from the inlet (except as noted in main text during optimization), using the PA800 plus UV detector set to collect absorbance at 214 nm. See Tables S14 and S15 in the ESM for instrument configuration details. New capillaries were conditioned twice using the method in ESM Table S16. Samples were analyzed using the method in ESM Table S17. At the end of each sequence, the capillary was prepared for storage using the method in ESM Table S18 then stored at room temperature with the ends dipped in DIUF water. Electropherograms were analyzed using the 32Karat software package (Sciex Separations) as described in the CZE data analysis section of the ESM.

## Results and discussion

### CIEF method development

**Fig. 1:**
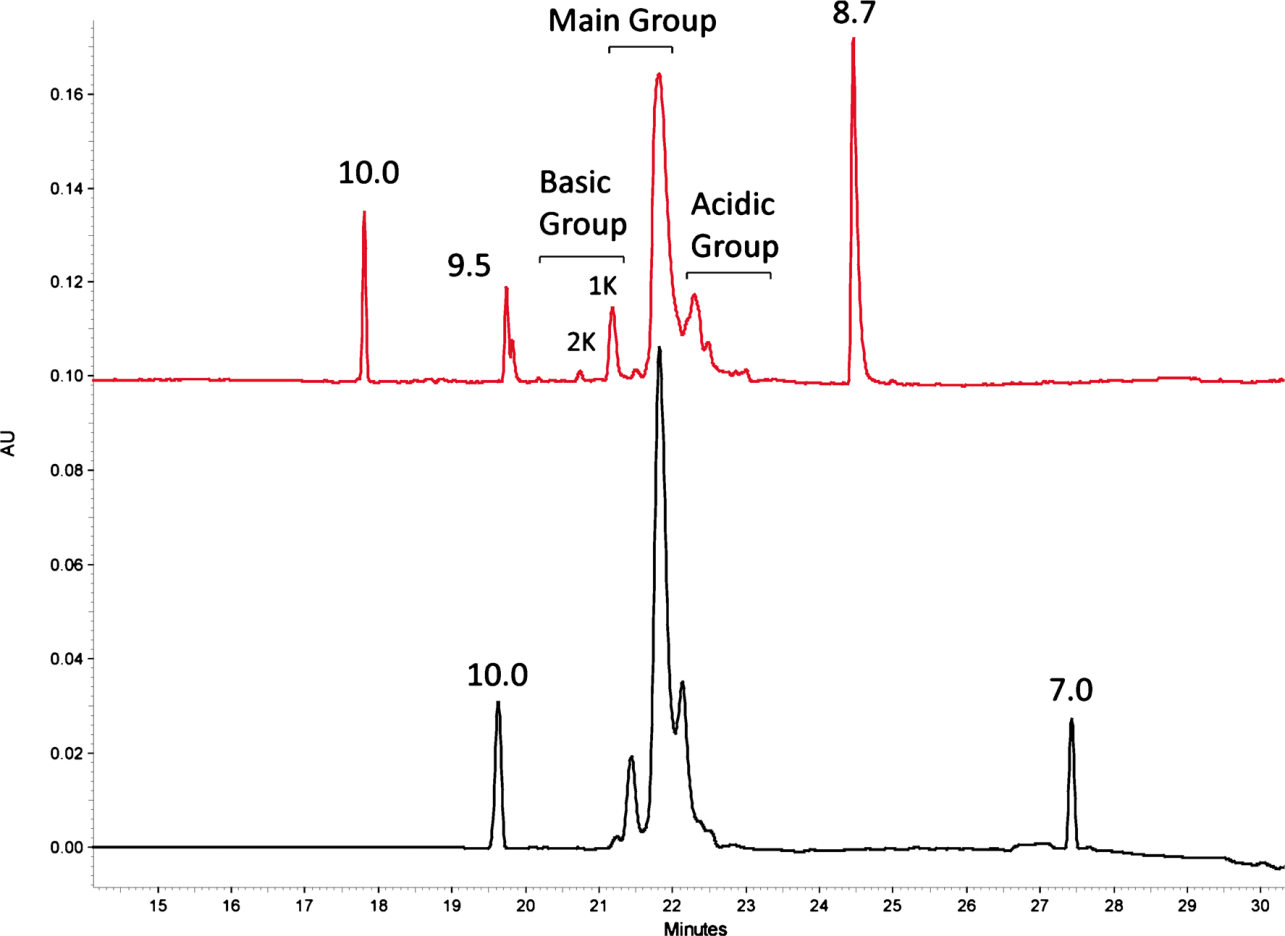
Comparison of starting (bottom) and optimized (top) CIEF profiles for PS 8670. Bottom trace: 3 mol/L urea, 100% Pharmalyte 3–10. Top trace: 1.5 mol/L urea, 25% Pharmalyte 3–10:75% Pharmalyte 8–10.5. 1 K: Basic variant with C-terminal lysine on one heavy chain. 2 K: Basic variant with C-terminal lysine on both heavy chain molecules

CIEF separates charge variants based on differences in isoelectric point [29]. CIEF has been employed in the biopharmaceutical industry for monitoring mAb charge variants using both fixed focal point detection and in an imaged format (iCIEF) where the entire capillary is imaged following the separation [11, 30]. A heterogeneous mAb sample is mixed with an ampholyte solution which forms a stable pH gradient when subjected to an applied electric field for a specified gradient focusing time. All charged species, including the mAb variants migrate in the applied field until they reach the pH where their net charge and electrophoretic mobility reaches zero; at this point, the pH is equal to the variant’s pI. Calibration against marker peptides of known pI spiked into the sample allow the apparent pI of the mAb variants to be estimated [31]. Detection of mAb variants is achieved via chemical mobilization during which time all species migrate past the detection window. A CIEF method was optimized for resolution and apparent pI determination of NISTmAb charge variants. The “out-of-the-box” and optimized charge profiles of the NISTmAb are shown in Fig. 1. The major basic species are C-terminal lysine variants, presumably resulting from incomplete processing by endogenous host-cell derived carboxypeptidase(s) present during production [12]; the peak labeled “1 K” retains the C-terminal lysine on one heavy chain, and the peak labeled “2 K” retains C-terminal lysines on both heavy chains. The acidic variants elute as a smear and contain deamidations, sialic acid variants, and other PTMs as discussed above.

Method development was based on the Sciex Separations CIEF analysis kit [28]. The Sciex platform method utilizes 200 mmol/L phospohoric acid as the anolyte, 300 mmol/L sodium hydroxide as the catholyte, and 300 mmol/L sodium hydroxide as the chemical mobilizer; each of which were determined to be suitable for NISTmAb separation and did not require further optimization. For the platform method, 50 to 100 μg of mAb sample in 10 μL of low salt buffer is mixed with a “master mix” (Table 1) consisting of cathodic and anodic stabilizers, separation gel buffer containing urea (to solubilize protein near pI), ampholytes and appropriate pI markers. Arginine and iminodiacetic acid are included as cathodic and anodic stabilizers, respectively, to prevent distortion of the pH gradient at the ends of the capillary and were used according to the manufacturer’s instructions [28, 32]. The optimization of urea concentration, ampholyte mixture, and appropriate pI markers are discussed in turn below.

**Fig. 2:**
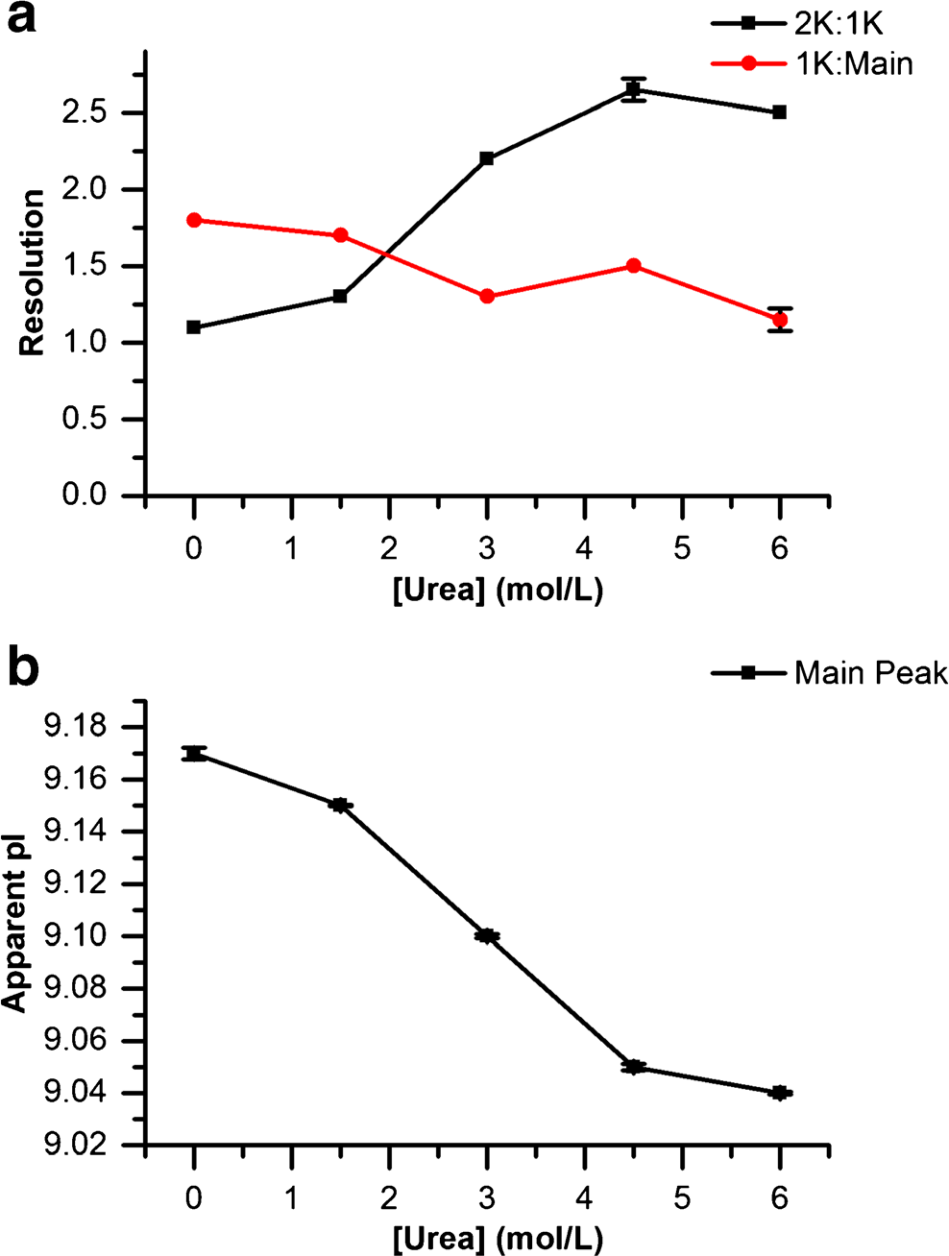
Effect of urea concentration on NISTmAb CIEF separation using Pharmalyte 3–10. (a) Effect of urea concentration on resolution of basic variants from each other (2 K:1 K) and on resolution of the 1 K variant from the main peak (1 K:Main). (b) Decrease in measured apparent pI of the NISTmAb main peak with added urea.  *n* = 2. Error bars indicate one standard deviation

Urea is often used in CIEF to improve protein solubility near the isoelectric point, however, urea concentration can affect resolution of charge variant species and the apparent pI; the manufacturer recommended concentration for this CIEF kit is 3 mol/L. Fig. 2 shows the effect of the concentration of urea in the CIEF gel buffer on resolution of NISTmAb charge variants using the manufacturer recommended Pharmalyte 3–10 as the ampholyte. The resolution between the 2 K and 1 K C-terminal lysine variants increased with increasing urea concentration, while the resolution between the 1 K variant and the main peak decreased slightly with added urea. However, the apparent pI of the main NISTmAb charge variant was significantly affected by added urea, probably due to urea-mediated denaturation. Based on these data, the urea concentration for NISTmAb analyses was lowered from 3 mol/L to 1.5 mol/L. This concentration allows sufficient resolution of the C-terminal lysine variants while minimizing pI deviations.

**Fig. 3:**
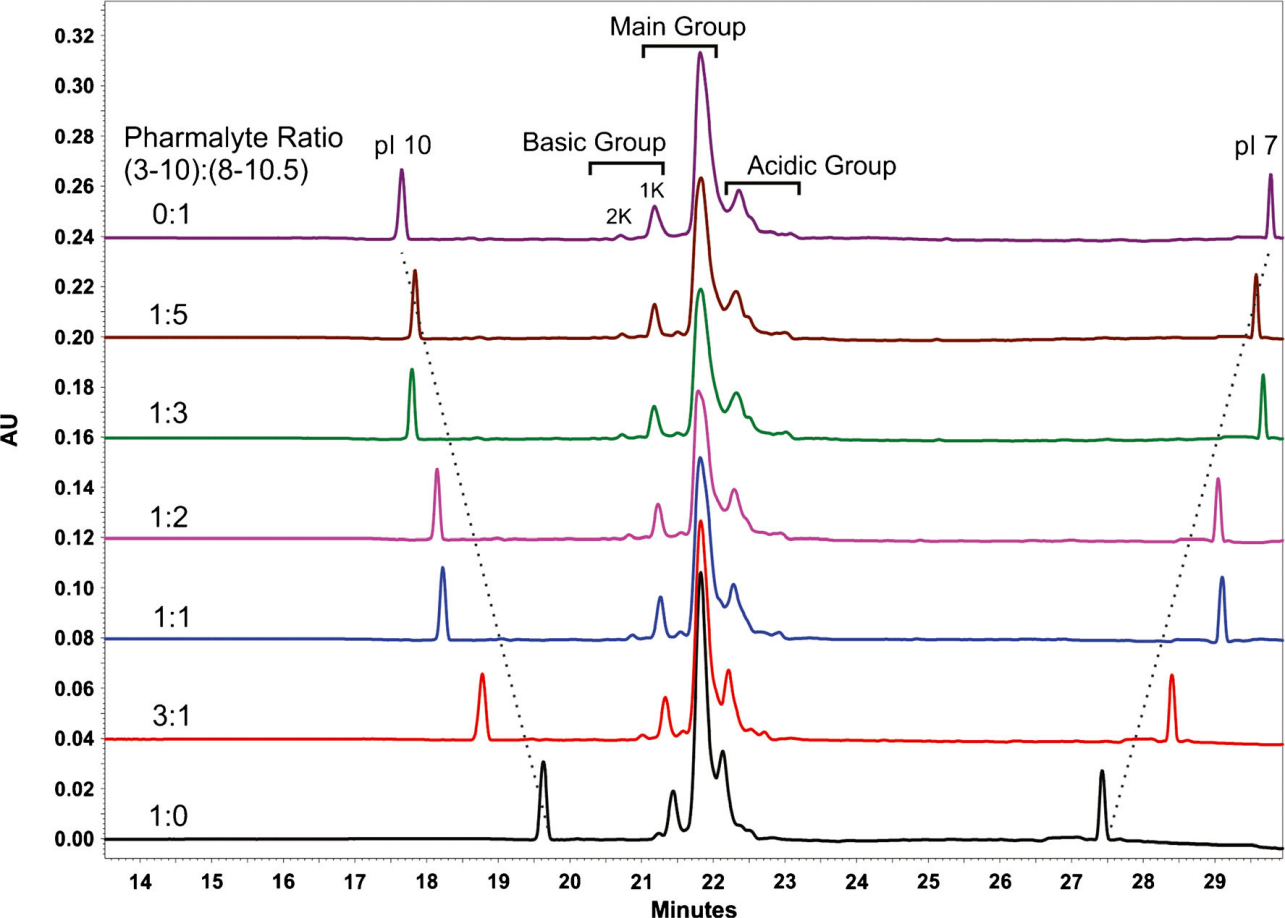
Effect of ampholyte composition on resolution of NISTmAb basic charge variants using 1.5 M urea CIEF gel buffer for the master mix

A major contributor to charge variant resolution in CIEF is the composition of the ampholyte mixture that forms the pH gradient. Ampholyte solutions can be chosen which cover a broad pH range (i.e. Pharmalyte 3–10) or which improve resolution within a narrow pH range (i.e. Pharmalyte 5–8 and 8–10.5) [33]. Various combinations of broad range Pharmalyte 3–10 and narrow range Pharmalyte 8–10.5 were tested for optimal resolution of NISTmAb charge variants; the total proportion of ampholyte in the sample was fixed at 4.8 vol.%. In general, decreasing the proportion of broad range Pharmalyte 3–10 (BR) relative to narrow range Pharmalyte 8–10.5 (NR) improves resolution of NISTmAb charge variants (Fig. 3). Beginning at a BR:NR ampholyte ratio of 1:2, the improvement in separation quality with added narrow range ampholyte levels off, until the separation quality decreases again at a ratio of 0:1. Based on the observation that the separation quality is essentially the same at BR:NR ratios of 1:2, 1:3, and 1:5, the middle value (1:3) was chosen as the optimal value with the expectation that this would result in a more robust separation. The optimal conditions for resolution of NISTmAb charge variants are therefore 1.5 mol/L urea and 25% Pharmalyte 3–10:75% Pharmalyte 8–10.5 (1:3 BR:NR).

**Fig. 4:**
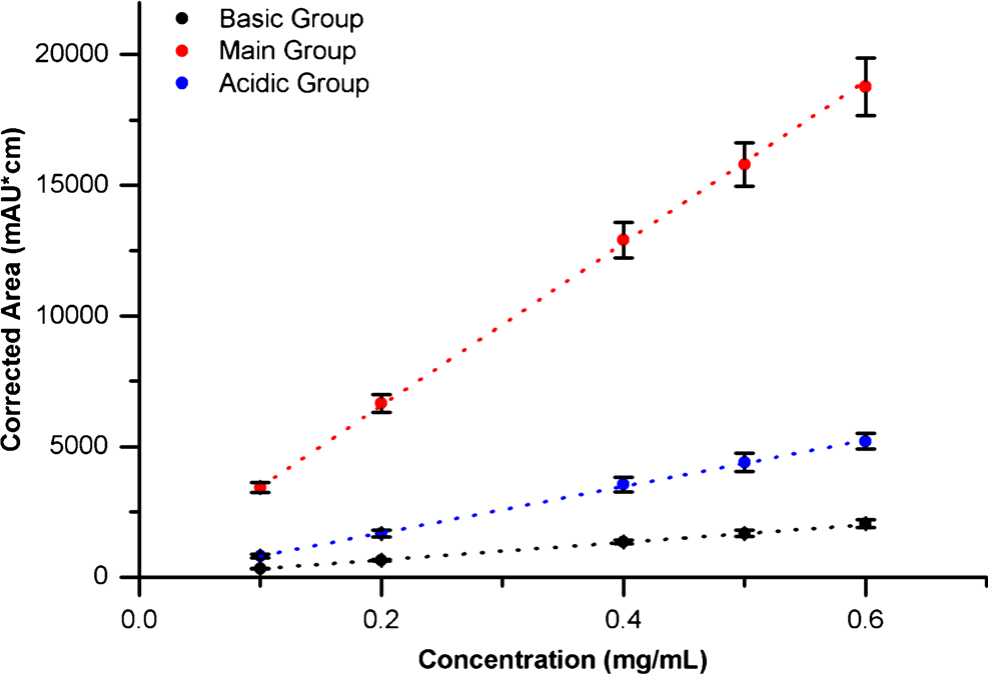
NISTmAb titration plots in CIEF by charge variant group (1.5 mol/L urea, 25% Pharmalyte 3–10:75% Pharmalyte 8–10.5). *n* = 3. Error bars indicate one standard deviation

The optimized method (1.5 mol/L Urea and 1:3 BR:NR Ampholyte) was further evaluated to determine suitability for qualification as a routine charge heterogeneity assay for NISTmAb quality control. The corrected area linear range was evaluated using a dilution series of 10 mg/mL NISTmAb PS 8670 in master mix (Fig. 4, see ESM Table S1 for details). The assay demonstrated excellent linearity for all charge groups (R-squared >0.98 and relative residual standard deviation <8%) from 0.1 to 0.6 mg/mL or 25 to 150% of the target loading concentration (See ESM linearity regressions section for description of relevant calculations). The limit of detection (LOD) and limit of quantification (LOQ) were estimated from the signal-to-noise ratio (SNR) of the 1 K peak at the target concentration (0.4 mg/mL, see ESM for relevant calculations). This target concentration was chosen because it is the maximum recommended by Sciex for their CIEF kit. The LOD and LOQ in units of mass were 3.6 (0.2) ng and 11.9 (0.7) ng of minor variant present in the sample, respectively. The mass-based values were converted to % relative abundance (RA) units at the target loading concentration (0.4 mg/mL), resulting in LOD and LOQ of 1.5 (0.1) % RA and 5.1 (0.3) % RA, respectively. See the ESM for relevant calculations. These values were higher than desired for a quantitative charge variant assay for NISTmAb quality control, despite satisfactory within-day precision and excellent resolution of charge variants (Tables 2 and 3). The relatively poor sensitivity of this assay is due to the necessity of detecting at 280 nm, rather than a lower wavelength at which the protein absorbs more strongly. Detection must be carried out at 280 nm because of high background from ampholyte absorption at lower wavelengths. Consequently, this assay was not qualified for routine charge heterogeneity monitoring of the NISTmAb.

**Table 2:** Analytical Figures of Merit of the Optimized CIEF Method for PS 8670

Parameter	Mean (SD)^a^	
Limit of Detection ^b^	3.6 (0.2) ng	1.5 (0.1) % RA at Target
Limit of Quantification ^b^	11.9 (0.7) ng	5.1 (0.3) % RA at Target
Linear Range (Main Peak)^b^	0.1 to 0.6 mg/mL	25 to 150% of Target
Resolution (1 K:Main)^b^	1.6 (0.1)	
Theoretical Plates (Main Peak)	8 × 10^4^	
Sample Consumption	240 ng	
Run Time per Sample	55 min	

^a^ *SD = standard deviation;* ^*b*^ *n = 3*

**Table 3:** Within-Day Precision of the Optimized CIEF Method for PS 8670

Measurand	Mean (SD)^a^	CV
Main Peak Migration Time (min)^b^	25.54 (0.20)	0.78%
Main Peak Apparent pI^b^	9.18 (0.01)	0.11%
Main Group RA (%)^b^	72.5 (0.4)	0.5%
Acidic Group RA (%)^b^	19.9 (0.5)	2.5%
Basic Group RA (%)^b^	7.6 (0.2)	2.6%

^a^ *SD = standard deviation;* ^*b*^ *n = 3*

The optimized CIEF assay is, however, highly useful as a characterization tool and for estimating apparent pI of NISTmAb charge variants. The pH gradient linearity using the optimized 1:3 BR:NR ampholye in the region of the NISTmAb pI was initially evaluated using the pI 7.0, 8.7, 9.5, and 10.0 markers. The pI 7.0 marker was shown to fall outside the linear region under these conditions, and was therefore not included in the final master mix. The linearity from pH 8.7 to pH 10 was acceptable and lead to the inclusion of pI 8.7, 9.5, and 10.0 pI markers as internal standards. Linear regression performed on migration time versus nominal pI on the CIEF linearity samples discussed above (*n* = 18 injections, pI markers 8.7, 9.5, and 10) resulted in an R-squared value of 0.98 and a relative residual standard deviation of 1.8% (See ESM linearity regressions section for description of relevant calculations). Similar plots for each individual injection resulted in an R-squared >0.98. The injection-specific pI calibration curves for the target concentration samples were used to determine an apparent pI for the NISTmAb main peak (Table 3). The assay resulted in good intra-day precision for pI determination and will be carried forward as a characterization tool for evaluating NISTmAb pI. As an alternative to CIEF for charge heterogeneity quality control measurements, a capillary zone electrophoresis (CZE) assay was developed.

### CZE method development

CZE separates analytes in free solution based on differences in electrophoretic mobility under an applied electric field in a buffer-filled capillary [29, 34]. The rate of migration in CZE is largely dependent upon net charge, with contributions from differences in hydrodynamic radius (related to molecular weight). The CZE charge heterogeneity assay proposed by He, et al. [20] was used as a starting point for further optimization. This method is gaining popularity as a rapid, inexpensive, high performance alternative to traditional CIEF-based charge variant assays, and was evaluated in a large inter-company study [19]. The method employs 400 mmol/L 6-aminocaproic acid (EACA) as a high concentration zwitterionic background electrolyte (BGE) supplemented with 2 mmol/L triethylenetetraamine (TETA) as a cationic capillary passivating reagent and 0.05% (*w*/*v*) hydroxypropylmethylcellulose (HPMC), a nonionic polymer which promotes protein solubilization and reduces adsorption. He, et al. found that a pH of 5.7 was optimal for separation of mAbs with a range of pIs from 7 to 9.5. No sample preparation beyond dilution to the desired concentration (0.5 to 1.5 mg/mL) with deionized water is required, provided the sample buffer is relatively low salt. Dilution with water promotes high separation efficiency through analyte stacking. Because the BGE is zwitterionic, a very high separation voltage (30 kV) may be applied even at high BGE concentrations without loss of efficiency due to Joule heating. The following separation parameters were evaluated for optimization of NISTmAb PS 8670 separation: 1) EACA and TETA concentrations, 2) capillary length, and 3) solubilizing agent.

**Fig. 5:**
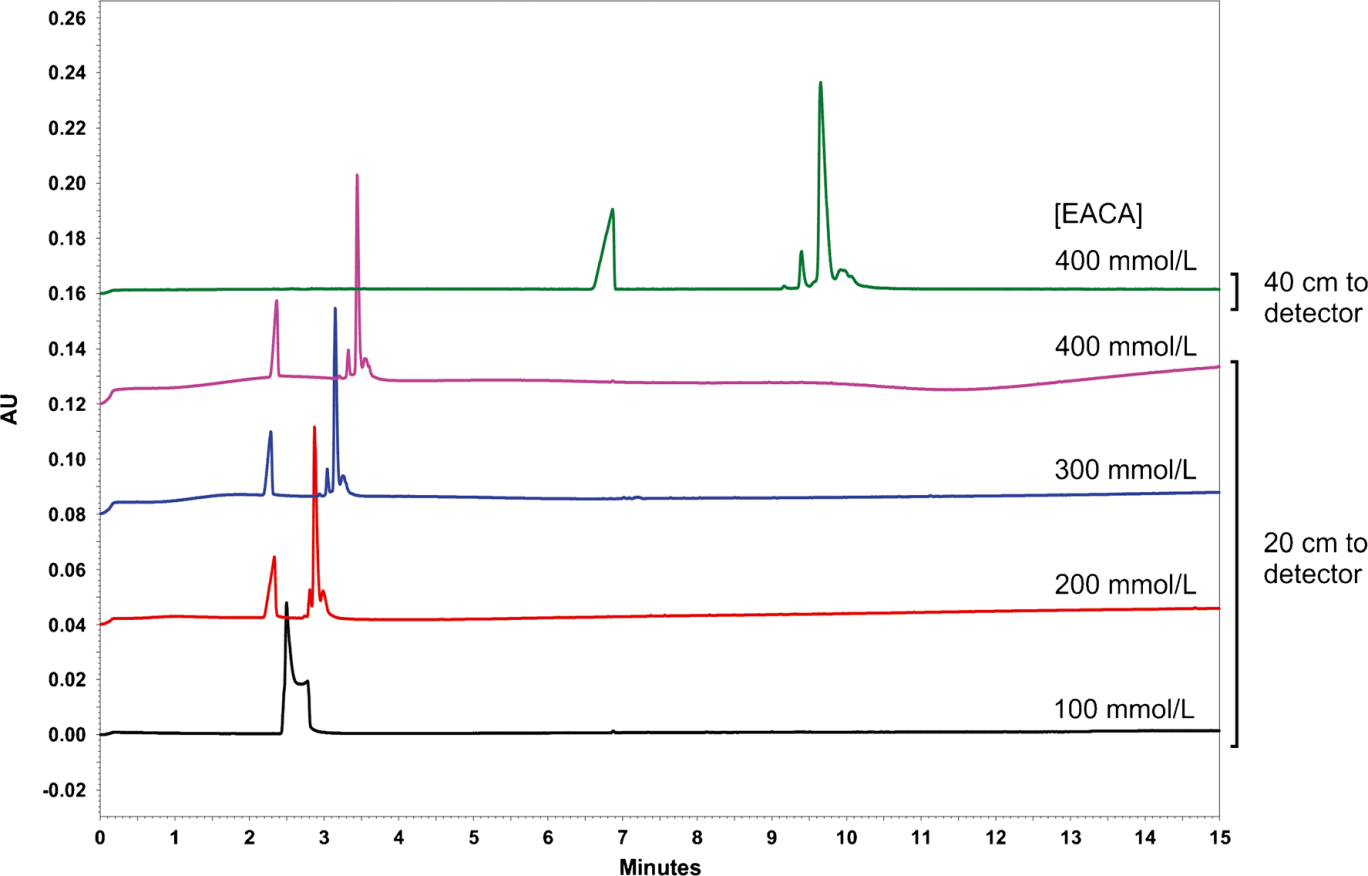
Effect of EACA concentration and effective capillary length on CZE separation; [TETA] = 2 mmol/L; [HPMC] = 0.05% (*w*/*v*); pH 5.7

**Fig. 6:**
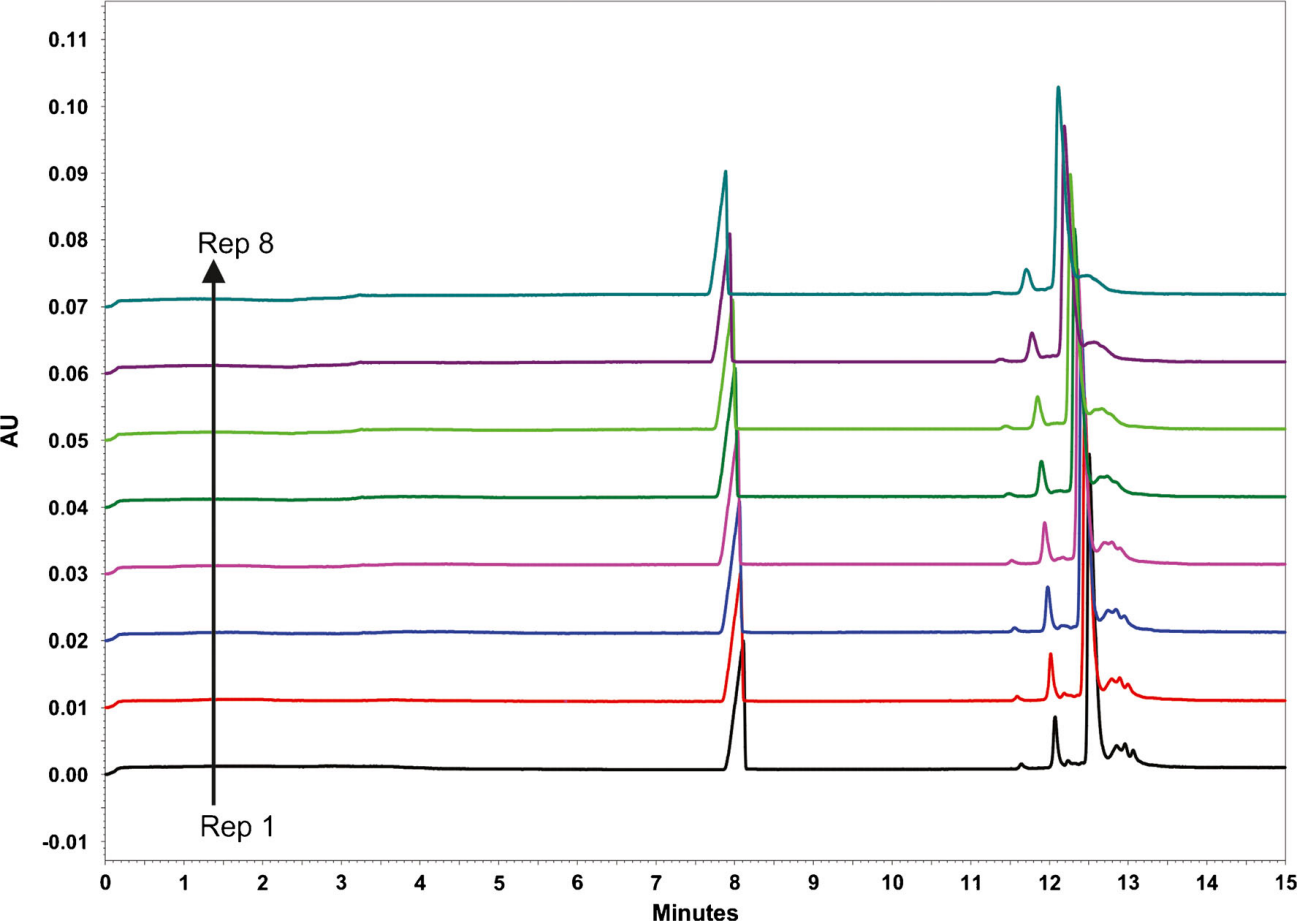
Replicate CZE analyses of Primary Sample 8670 using 400 mmol/L EACA, 2 mmol/L TETA (pH 5.7), 0.05% (w/v) HPMC. Separation efficiency decreases with repeated injections

Starting from the published BGE (400 mmol/L EACA, 2 mmol/L TETA, 0.05% (*w*/*v*) HPMC, pH 5.7), the BGE was optimized to improve the robustness and resolution of the NISTmAb charge variant separation. No significant improvement was observed by varying the TETA concentration from 0.2 mmol/L to 2 mmol/L. Further increase to 10 mmol/L resulted in total and irreversible loss of separation, presumably due to incomplete regeneration of the capillary surface between injections. The TETA concentration was therefore fixed at 2 mmol/L as it was assumed that the greater excess of passivating reagent would result in a more robust method. Next, the concentration of EACA was optimized at 2 mmol/L TETA (pH 5.7) and 0.05% (w/v) HPMC (Fig. 5, bottom traces). Charge variant resolution increased with increasing EACA concentration from 100 mmol/L to 400 mmol/L; therefore, the concentration of EACA was set at 400 mmol/L. The best resolution of NISTmAb charge variants was observed with the TETA and EACA concentrations as published. In order to further increase charge variant resolution, the capillary length and length to detector were increased from 30.5 cm capillary (20 cm to detector) to 50.5 cm (40 cm to detector). The increase in capillary effective length increased the separation time from ≈5 min to ≈15 min, but also significantly improved resolution of NISTmAb charge variants by increasing the total separation space (Fig. 6, top trace). Therefore, 50.5 cm capillaries (40 cm to detector) were used going forward.

**Fig. 7:**
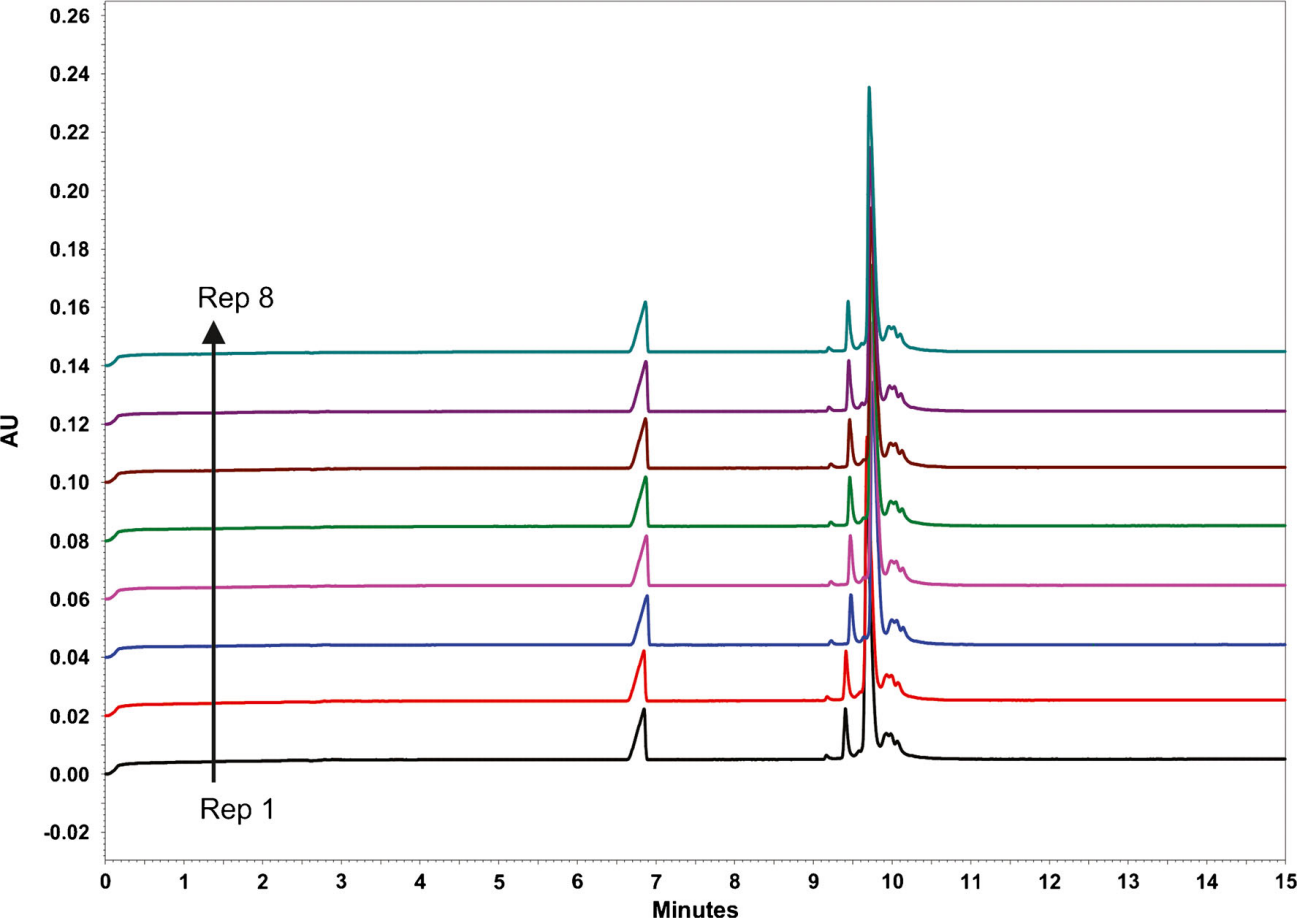
Replicate CZE analyses of Primary Sample 8670 using 400 mmol/L EACA, 2 mmol/L TETA (pH 5.7), 0.03% (w/v) Tween™ 20

**Fig. 8:**
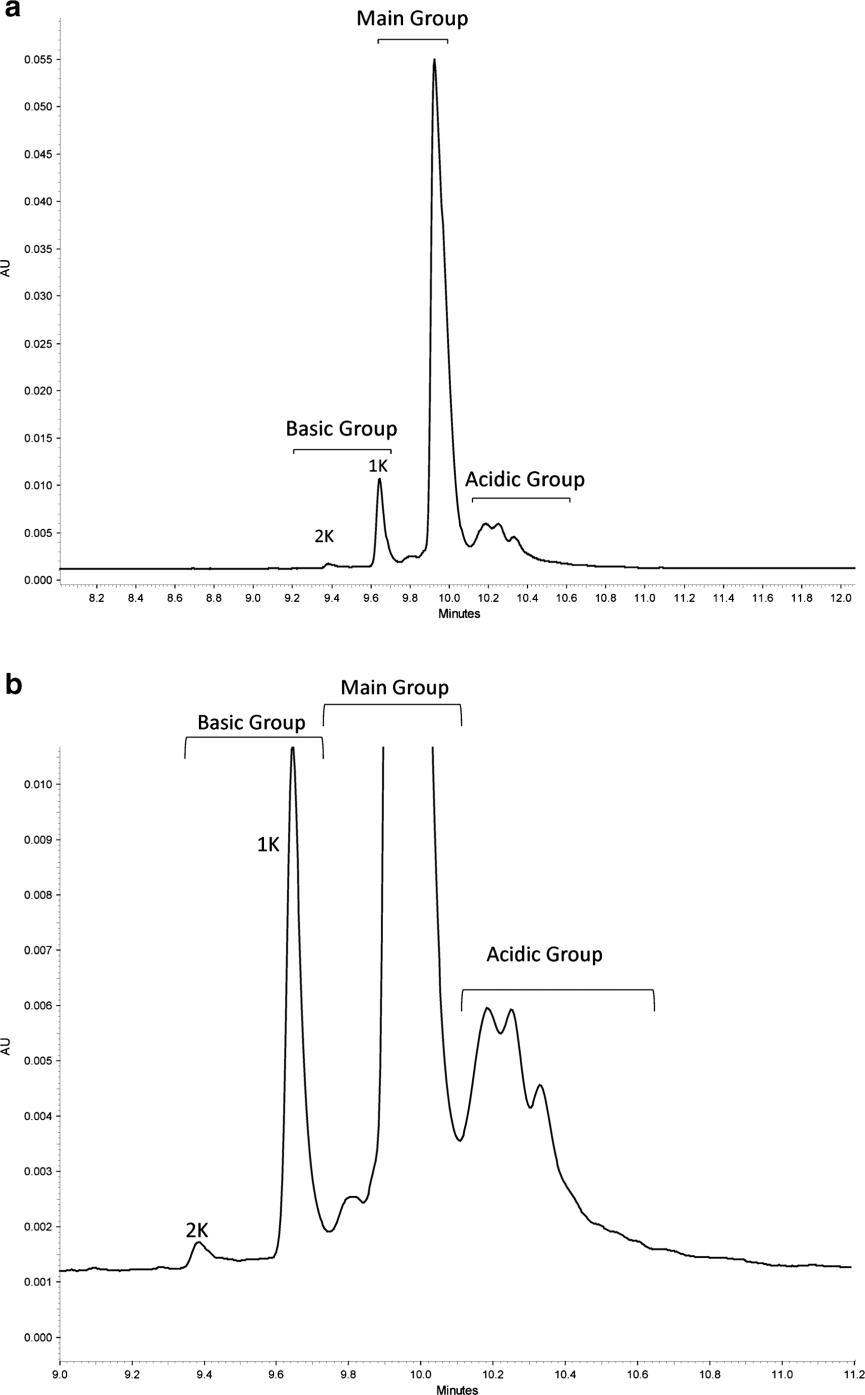
Optimized CZE separation of NISTmAb charge variants (a) full scale and (b) expanded view (400 mmol/L EACA, 2 mmol/L TETA (pH 5.7), 0.03% (w/v) Tween™ 20)

During repeatability testing of the HPMC-containing BGE system, the separation quality was observed to degrade over time and was not recoverable by re-conditioning (Figure 6). Brief rinsing with 100 mmol/L sodium hydroxide lead to temporary recovery of separation efficiency, but repeated base treatments eventually lead to total separation loss, presumably due to exposure of deprotonated silanols on the capillary surface leading to protein adsorption. Anecdotal evidence pointed to degradation of HPMC in the EACA buffer as the cause of gradual separation loss. HPMC and EACA/TETA solutions were prepared separately and mixed fresh each day. As this did not mitigate separation loss over time, poor stability of HPMC was suspected. Since HPMC proved to be difficult to prepare and handle, Tween™ 20 was evaluated as an alternative protein solubilizing agent. Tween™ 20 is commonly employed in antibody assays to minimize non-specific protein binding. BGE was prepared with 400 mmol/L EACA, 2 mmol/L TETA (pH 5.7) and 0.03% (*w*/*v*) Tween™ 20 and tested in a 50.5 cm (40 cm to detector) capillary. The applied voltage was maintained at 30 kV, the maximum achievable by this instrument. As is evident in Fig. 7, the EACA/TETA/Tween™ 20 buffer yielded excellent separation of Primary Sample 8670 charge variants in a highly rapid and reproducible manner. Separation quality did not degrade even after many uses (>50) of the same capillary. Therefore, these separation conditions were chosen for further testing. The optimized charge profile of the NISTmAb is given in Fig. 8.

### CZE method qualification

The optimized CZE assay was determined to be suitable for further qualification. The qualification plan adopted here, described in [27], is based on ICH Q2(R1) and has been scaled to meet the needs of the NISTmAb RM 8671 Lifecycle Management Plan. Briefly, the method was evaluated for acceptable linear range, limit of detection (LOD), limit of quantification (LOQ), specificity, and limited intermediate precision as described in the ESM. The analytical figures of merit of the optimized method are summarized in Table 4.

**Table 4:** Analytical Figures of Merit for CZE

Parameter	Mean (SD)^a^	
Limit of Detection ^b^	0.044 (0.012) ng	0.2 (0.1) % RA at Target
Limit of Quantification ^b^	0.150 (0.039) ng	0.7 (0.2) % RA at Target
Linear Range (Main Peak) ^b^	0.25 to 2.5 mg/mL	17 to 170% of Target
Resolution (1 K:Main) ^b^	0.9 (0.001)	
Theoretical Plates (Main Peak) ^b^	6 × 10^4^	
Sample Consumption	20 ng	
Run Time per Sample	25 min	

^a^ *SD = standard deviation;* ^b^ *n = 3*

### CZE method linearity

**Fig. 9:**
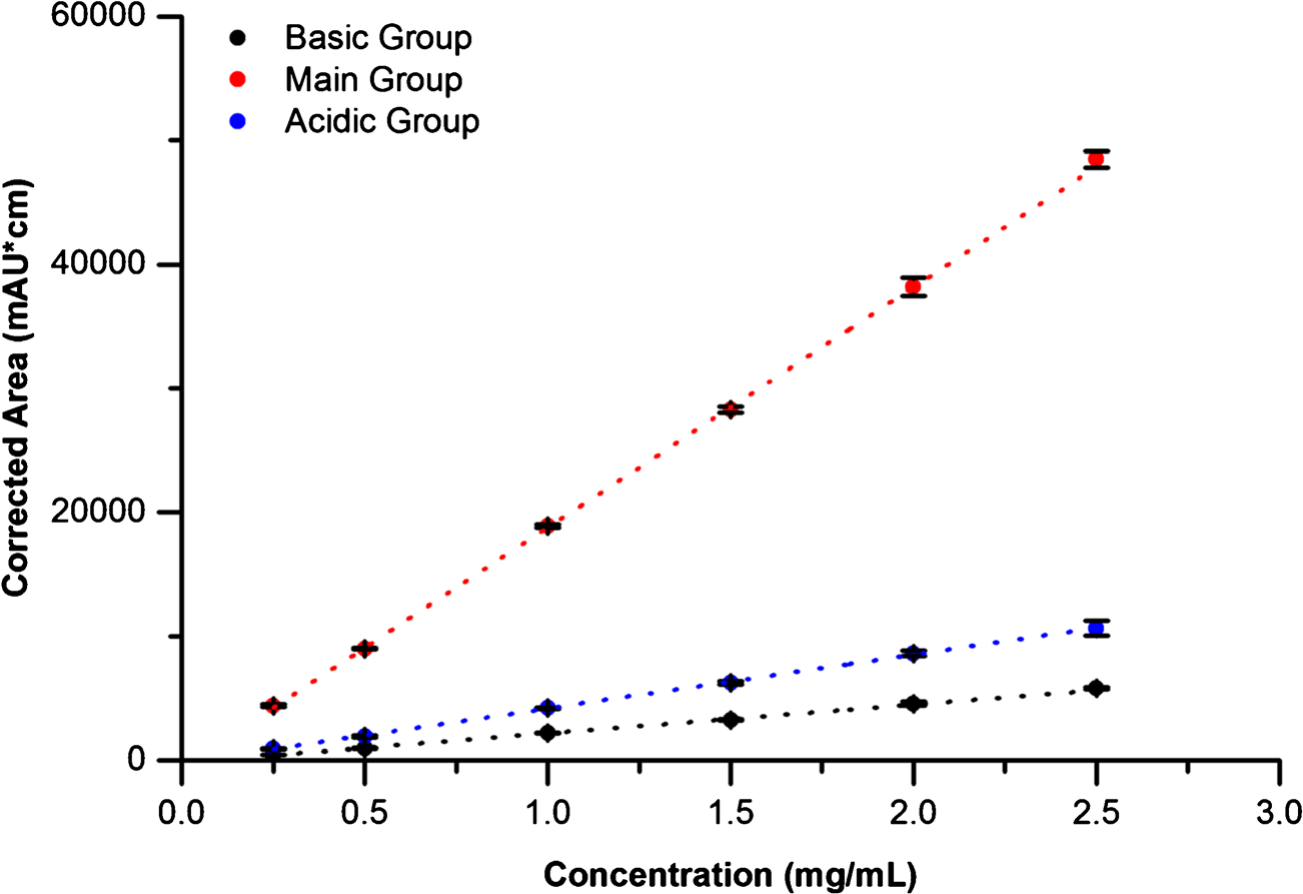
NISTmAb titration plots in CZE by charge variant group (400 mmol/L EACA, 2 mmol/L TETA (pH 5.7), 0.03% (w/v) Tween™ 20). *n* = 3. Error bars indicate one standard deviation

A sample of Primary Sample 8670 in formulation buffer (10 mg/mL) was diluted to 2.5 mg/mL in type 1 deionized ultrafiltered water (DIUF). Serial dilutions of the 2.5 mg/mL sample were prepared down to 0.025 mg/mL. The assay was found to be linear with respect to Primary Sample 8670 loading concentration for all charge variant groups from 0.25 mg/mL to 2.5 mg/mL (R^2^ > 0.99 and relative residual standard deviation <5%, see Table S6 in the ESM). This linear range covers 17% to 170% of the target loading concentration of 1.5 mg/mL (Fig. 9). It should be noted that Figure 9 plots mean corrected area, however linearity assessment was performed using each individual data point as described in the ESM to allow appropriate statistical fit evaluation.

### CZE limit of detection/limit of quantification

The limits of detection and quantification (LOD and LOQ) of the method were estimated from the signal-to-noise (SNR) value of the 2 K basic variant in the 1.5 mg/mL sample (the target loading concentration). The LOD and LOQ in units of mass were 0.044 (0.012) ng and 0.150 (0.039) ng (SD) of minor variant present in the sample, respectively. The mass-based values were converted to % relative abundance (RA) units at the target loading concentration (1.5 mg/mL), resulting in LOD and LOQ of 0.2 (0.1) % RA and 0.7 (0.2) % RA, respectively. See the ESM for relevant calculations. This is an improvement over the optimized CIEF assay, indicating the advantage of detection at 214 nm over 280 nm for sensitive analysis of minor charge variants.

### CZE Specificity

**Fig. 10:**
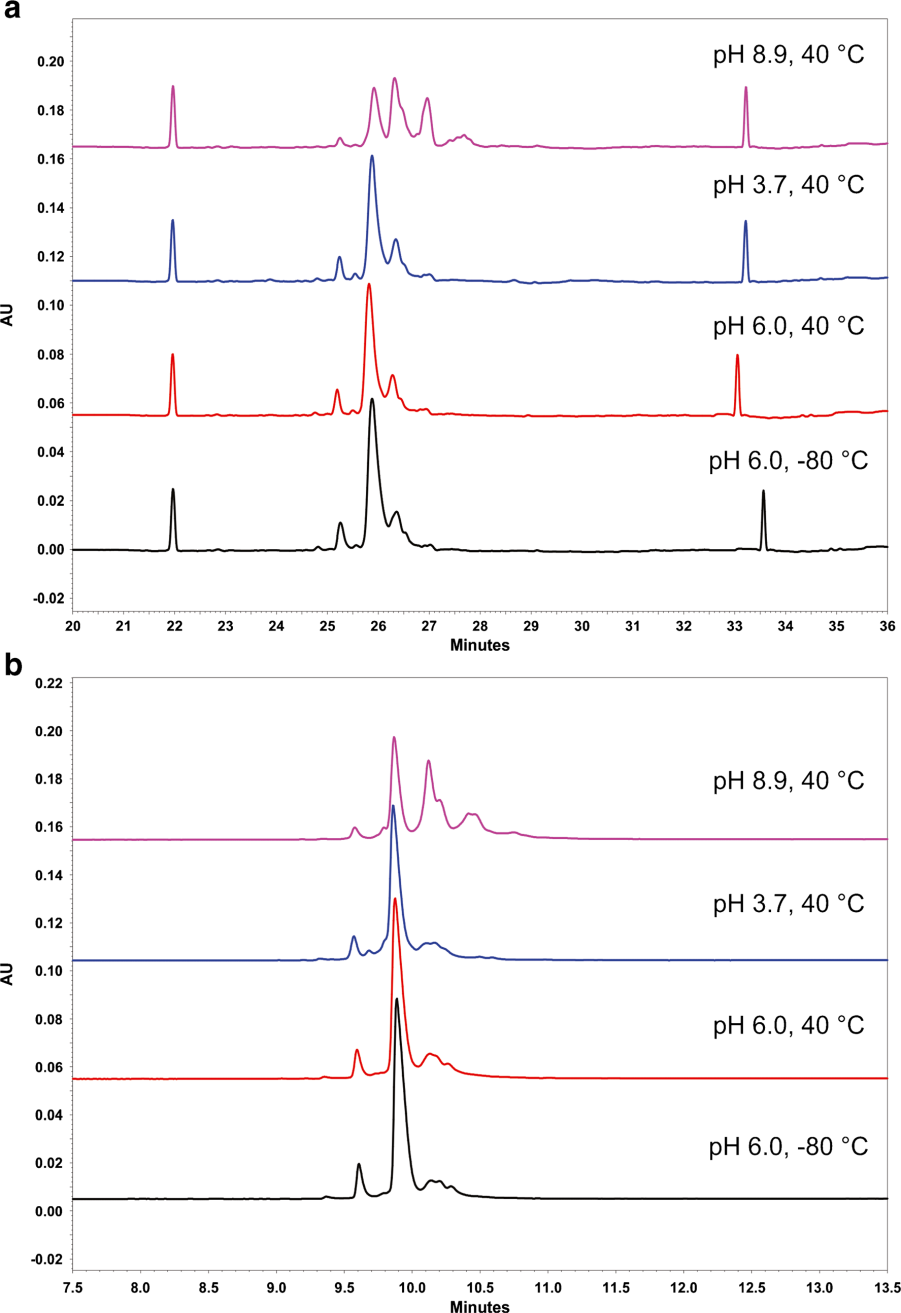
Comparison of optimized CIEF (a) and optimized CZE (b) profiles of NISTmAb subjected to pH and thermal stress

The specificity of the assay with respect to potential matrix interference was assessed by verifying the absence of interfering peaks in the blank (formulation buffer only). Only one peak is present in the blank, attributed to the L-His component of the formulation buffer. This peak is labeled “Excipient” in Fig. 7. The total carryover of the method was confirmed to be negligible based on no detectible signal (other than L-Histidine peak) for a blank injection immediately following a sample injection. Specificity with respect to potential degradants was evaluated using forced degraded material. The assay was applied to analysis of Primary Sample 8670 subjected to stress conditions designed to induce varying levels of charge heterogeneity. Primary Sample 8670 was buffer exchanged into buffers of various pH (formulation buffer, pH 6.0; acetate buffer, pH 3.7; phosphate buffer, pH 8.9) and incubated at 40 °C for 8 days prior to analysis. The control sample was maintained in formulation buffer at −80 °C until analysis. As expected, the lower pH stress conditions generated minor changes in charge variant profile, while the high pH condition caused extensive conversion of the main charge variant to various acidic variants (Figure 10b). Therefore, this assay is stability indicating and may be used to quantify changes in charge variant distribution of the Primary Sample 8670. Comparison of CZE profiles of stressed PS 8670 with CIEF profiles of similar samples (Figure 10a) demonstrates comparability of CZE to CIEF for stability indication of the NISTmAb.

### CZE intermediate precision

The intermediate precision of the method was estimated from 54 injections of PS 8670 over three capillaries/buffer preparations and six days. The injection sequence for each day followed the form: Blank─IQ─8670 Prep #1 × 3─IQ─8670 Prep #2 × 3─IQ─8670 Prep #3 × 3─IQ─Blank. Here, IQ is the instrument qualification control prepared by diluting 10 μL of the pI 10.0 peptide marker to 1/10X with 90 μL of DIUF water and mixing. The parameters recorded for each sample are given in Table S5 in the ESM. The intermediate precision is defined here as the combined standard uncertainty (*u*_*c*_), at the level of one standard deviation, of all the measurements for a given parameter (Table 5); refer to the ESM and the discussion in [27] in this series for detailed information on the calculation of this uncertainty. The method precision is acceptable for the purposes of NISTmAb charge heterogeneity monitoring, with CVs less than 5% for all parameters.

**Table 5:** Intermediate Precision of Optimized CZE Method

Parameter	Mean ± *u*_*c*_^a^	CV
Instrument Qualification Standard (IQ)		
IQ Standard Migration Time (min)	5.79 ± 0.06	1.0%
PS 8670		
Main Peak Migration Time (min)	9.67 ± 0.17	1.8%
Main Group RA (%)	74.7 ± 0.3	0.5%
Acidic Group RA (%)	16.8 ± 0.4	2.4%
Basic Group RA (%)	8.5 ± 0.3	3.3%

^a^*Stated uncertainty represents the intermediate precision reported as a combined standard uncertainty, at a level of one standard deviation, based on ANOVA analysis as described in* ESM *(n = 54)*

### CZE performance criteria

The IQ standard and PS 8670 will be used to evaluate system performance and system suitability, respectively, during NISTmAb RM 8671 value assignment [35]. The performance criteria for the method were set for each parameter based on the measured intermediate precision. These criteria are useful for ensuring that the analytical method is in control, thus establishing confidence in the data acquired using the method. Performance criteria for the optimized CZE method can be found in the ESM.

### CZE accuracy

The accuracy of the method was inferred from measured linearity, specificity, and precision as provided for in ICH Q2(R1) [36]. The accuracy of the method is further supported by comparison to orthogonal methods including the CIEF method developed here and historical data as discussed in detail below.

### Comparison of methods

Two methods for measuring mAb charge heterogeneity were optimized using the NISTmAb as a test molecule. CIEF is a well-established method commonly employed in the biotherapeutics industry, while the CZE method is a recent innovation that is steadily gaining traction. Comparison on the NISTmAb highlights the strengths and weaknesses of each method. CIEF affords excellent resolution of charge variants and provides information about their apparent pI. However, CIEF suffers from poor sensitivity. Additionally, the CIEF assay is expensive and time consuming, both in terms of sample preparation and actual instrument run time. CZE does not inform as to apparent pI of the analytes, but it provides comparable resolution of charge variants and stability indication, superior sensitivity, and excellent precision in less than half the time of a CIEF assay. The reagents for CZE are comparatively inexpensive, and sample preparation is minimal. The simplicity of the CZE assay contributes to its robustness and makes it an attractive alternative for routine quality monitoring, but it cannot fully replace CIEF as a characterization tool. This analysis highlights the utility of the NISTmAb as a platform for evaluating established and new analytical technologies in an open-source environment which facilitates and accelerates innovation.

### Comparison to historical values

The work presented here builds upon a previously published crowd-sourced large scale characterization of PS 8670 [11] in which established biotherapeutic research laboratories were asked to analyze the NISTmAb using their platform analytical methods. The NISTmAb was analyzed by multiple charge sensitive assays including CZE and CIEF; the results of those assays are compared to those measured here in Table 6.

**Table 6:** Comparison of PS 8670 Charge Heterogeneity by Method

	CIEF (book)	CIEF (optimized)^a^	iCIEF (book)	CZE (book)	CZE (qualified)^b^	CEX (book)
Main Group (%)	66.6	72.5 (0.4)	66.6	67.5	74.7 ± 0.3	72.6
Acidic Group (%)	24.1	19.9 (0.5)	24.2	21.6	16.8 ± 0.4	14.4
Basic Group (%)	9.3	7.6 (0.2)	9.2	10.9	8.5 ± 0.3	13.0
Apparent pI	–	9.18 (0.01)	9.3	–	–	–

^a^ *Stated uncertainty represents one within-day standard deviation (n = 3);* ^*b*^ *Stated uncertainty represents the intermediate precision reported as a combined standard uncertainty, at a level of one standard deviation, based on ANOVA analysis as described in the ESM (n = 54)*

The historical charge heterogeneity values (“book” values) are internally consistent across the three CE-based methods performed in the same laboratory. The charge heterogeneity values measured in the current work are also largely internally consistent, although the relative abundance of acidic variants was slightly higher in CIEF, reflecting different resolution of acidic variants in this method. The charge heterogeneity measured in this work by optimized methods is consistently higher than the historical values, with the exception of cation exchange chromatography (CEX). Careful examination of the electrophoretic data suggests that this is due to differences in data analysis and integration parameters (see ESM for full discussion). The choice of data analysis software and integration parameters has a significant effect on the results of electrophoretic assays, especially when baseline resolution is not achieved as is the case in mAb charge assays. The analysis parameters chosen for this work were optimized for reproducibility over large data sets at multiple loading concentrations. This approach is based on the desire to minimize the need for manual integration fixes. In particular for CIEF, the thresholding values were optimized to control false positives from the high ampholyte signal, at the expense of sensitivity. Less conservative thresholding in CIEF results in calculated charge heterogeneity values comparable to the historical data (see Table S3 in the ESM). Integration parameters were rationally selected to afford the highest consistency across our longitudinally acquired data sets. Interestingly, the charge heterogeneity values measured in-house by CZE using the previously published method agree with the in-house measurement using the optimized qualified method. This further highlights the influence of data analysis on reported results and the need to include data analysis and integration parameters in any method qualification plan.

The values for charge variant relative abundance reported here should not be taken as absolute values, but rather as method-specific reference values. Changes to the method, including data analysis parameters, may change the measured charge heterogeneity. Furthermore, the NISTmAb serves as a useful tool for evaluating the effect of data analysis on measured purity values and may contribute to a broader discussion about best practices for integration of CE data.

## Conclusions

The assays described here were optimized to be fit for their intended purpose. The CIEF assay is a valuable characterization tool which affords information about charge variant apparent pI which is not available from CZE. However, the CZE assay was found to be suitable for qualification as a routine quality monitoring assay due to excellent sensitivity, speed, simplicity, specificity, and intermediate precision. Based on the results of this work, CZE was selected for longitudinal quality monitoring of the NISTmAb Reference Material charge heterogeneity. Ongoing and future work may include optimized orthogonal methods, developed at NIST and/or in stakeholder laboratories, for assessing NISTmAb charge heterogeneity. The NISTmAb will serve as a common test case through which these orthogonal methods can be compared and refined across many laboratories and analysts to promote fuller understanding of the methods and best practices for their use.

## Electronic supplementary material

ESM 1 (PDF 2.44 mb)
