# Supplementary material for: Qualification of NISTmAb charge heterogeneity control assays
Source: Anal Bioanal Chem. 2018 Feb 9;410(8):2079–93. doi: 10.1007/s00216-017-0816-6 (PMC5830499; doi:10.1007/s00216-017-0816-6)
Supplement: Supplementary file 1 — (PDF 2.44 mb) [file 216_2017_816_MOESM1_ESM.pdf]

Analytical and Bioanalytical Chemistry

Electronic Supplementary Material

Qualification of NISTmAb charge heterogeneity control assays

Abigail Turner, John E. Schiel

Buffer and Stress Sample Preparation

Formulation Buffer Preparation. Mass measurements for solution preparation were performed on a Mettler Toledo AL54 balance (calibrated annually). Formulation buffer (12.5 mmol/L L-histidine/12.5 mmol/L L-histidine HCl, pH 6.0) was prepared as follows: 1) Weighed out 1.3129 g histidine monohydrochloride monohydrate and 0.9704 g L-histidine and diluted with ≈450 mL type 1 deionized ultrafiltered water (DIUF) that was obtained from an ultrapure water system fed with reverse osmosis water and filtered through a 0.2 µm filter; 2) while recording pH with a calibrated pH meter, adjusted pH by drop-wise addition of 1 mol/L hydrochloric acid to 6.00±0.02; 3) transferred to a 500 mL volumetric flask, rinsed beaker with DIUF water and adjusted flask volume to 500 mL using the rinse water; 4) sterile filtered using a 0.22 µm cellulose acetate membrane into a sterile plastic bottle; 5) stored at 2 °C to 8 °C.

Low pH Stress Buffer. Acetate buffer (50 mmol/L, pH 3.7) for low pH stress experiments was prepared from 200 mmol/L stock solutions of acetic acid and sodium acetate. The acetic acid stock was prepared as follows: 1) Dispensed 5.7 mL glacial acetic acid into a 500 mL class A volumetric flask containing ≈400 mL DIUF water; 2) diluted to 500 mL using DIUF water; 3) capped and inverted flask 10x to mix; 4) transferred to a plastic bottle and stored at room temperature. The sodium acetate stock solution was prepared as follows: 1) Weighed out 13.61 g sodium acetate (anhydrous); 2) dissolved in ≈450 mL DIUF water in a 500 mL class A volumetric flask; 3) diluted to 500 mL using DIUF water; 4) transferred to a plastic bottle and stored at room temperature. The final 50 mmol/L acetate buffer was prepared by mixing 2.5 mL of 200 mmol/L sodium acetate and 47.5 mL of 200 mmol/L acetic acid in a 50 mL plastic tube. The final solution was found to have a pH of 3.67 ± 0.02. The final solution was stored at room temperature.

High pH Stress Buffer. Phosphate buffer (50 mmol/L, pH 8.9) for high pH stress experiments was prepared as follows: 1) Weighed out 0.8924 g sodium phosphate dibasic dihydrate; 2) dissolved in ≈45 mL DIUF water; 3) adjusted pH to 8.9 using 0.1 mol/L sodium hydroxide and 0.1 mol/L hydrochloric acid; 4) quantitatively transferred to a 50 mL class A volumetric flask; 5) adjusted volume to 50 mL using DIUF water; 6) capped flask and inverted 10x to mix contents; 7) sterilized by passing through a 0.22 µm filter; 8) checked pH and adjust to 8.89 ± 0.02. Stored at room temperature.

Preparation of Stressed Samples. Portions of PS 8670 were buffer exchanged into one of the following buffers using Zeba 10K MWCO spin columns: formulation buffer; 50 mmol/L acetate, pH 3.7; or 50 mmol/L phosphate, pH 8.9. Samples were transferred to tightly capped 0.5 mL LoBind tubes and incubated at 40 °C for 8 d. A control sample in histidine buffer was stored at -80 °C until analysis. On day 8, samples were removed from the incubator and placed at -20 °C until analysis.

Capillary Isoelectric Focusing (CIEF)

CIEF Linearity/LOD/LOQ Sample Preparation. Samples for linearity/LOD/LOQ studies were prepared in a dilution series as follows. Master mix was prepared as in Table 1 according to the optimized method but scaled 23 fold. The master mix was vortexed 3 x 30 s on high before use. A master mix blank (3 reps) was prepared by mixing 54 µL of formulation buffer (12.5 mmol/L L-histidine, 12.5 mmol/L L-histidine HCl, pH 6.0) with 846 µL of master mix. A 0.6 mg/mL PS 8670 in master mix stock (in triplicate) was prepared by mixing 54 µL of 10 mg/mL PS 8670 in formulation buffer with 846 µL of master mix. A dilution series was prepared in triplicate from these solutions according to Table S1.

Table S1 Dilution Series of PS 8670 for CIEF Linearity/LOD/LOQ Determination

**Concentration (mg/mL)**	**Volume of mAb in Master Mix Stock**	**Volume of Master Mix Blank**
0.5	600 µL of 0.6 mg/mL	120 µL
0.4 (target concentration)	400 µL of 0.5 mg/mL	100 µL
0.2	200 µL of 0.4 mg/mL	200 µL
0.1	125 µL of 0.2 mg/mL	125 µL

CIEF Data Analysis. Electropherograms were analyzed using the 32Karat software package (Sciex Separations) using optimized integration parameters (Table S13). Retention times and corrected peak areas were recorded as in Table S2.

Table S2 Measured and Calculated Assay Results for CIEF Electropherograms

**Sample**	**Measurand Recorded**	**Calculated Parameters**
Blank	Presence of new peaks (Y/N)	n/a

IQ	PI peptides (8.7, 9.5 10.0) migration time (min)	n/a
8670	Main peak migration time (min)<br>Main peak group corrected area<br>Basic peaks (2K peak, 1K peak) corrected areas<br>Acidic peak group corrected area	Charge purity (%)<br>Basic Variants RA (%)<br>Acidic Variants RA (%)

Calculated parameters were derived from the recorded data using the following equations. Main group relative abundance was calculated according to equation S1:

$$Main\ Peak\ RA\ (\%) = \frac{CA_{main\ group}}{CA_{main\ group} + CA_{2K} + CA_{1K} + CA_{acidic\ group}} \times 100 \qquad \text{(S1)}$$

where $CA_{main\ group}$ is the corrected area of the main group, CA_{2K} is the corrected area of the 2K basic variant peak, CA_{1K} is the corrected area of the 1K basic variant peak, and $CA_{acidic\ group}$ is the corrected area of the acidic group as calculated by the 32Karat software. The relative abundance (RA) of acidic variants was calculated according to equation S2:

$$Acidic\ Variants\ RA\ (\%) = \frac{CA_{acidic\ group}}{CA_{main\ group} + CA_{2K} + CA_{1K} + CA_{acidic\ group}} \times 100\% \qquad \text{(S2)}$$

The relative abundance of basic variants was calculated using equation S3:

$$Basic\ Variants\ RA\ (\%) = \frac{CA_{2K} + CA_{1K}}{CA_{main\ group} + CA_{2K} + CA_{1K} + CA_{acidic\ group}} \times 100\% \qquad \text{(S3)}$$

A plot of pI marker peptide migration time (pI 8.7, 9.5, and 10.0) versus nominal pI was plotted for each individual injection. These plots were fit to linear regressions of the form Y=m*X+b in order to calculate the apparent pI value of NISTmAb charge variants based on migration time. The apparent pI of NISTmAb charge variants was calculated as in equation S4:

$$pI_{apparent} = \frac{t_{m,NISTmAb} - b}{m} \quad \text{(S4)}$$

where $t_{m,NISTmAb}$ is the migration time of the NISTmAb charge variant of interest, b is the y-intercept of the linear fit equation, and m is the slope of the linear fit equation.

Linearity Regressions. Plots of migration time versus nominal pI (pI 8.7, 9.5, and 10.0) or of corrected area versus loading concentration (similar to Figure 4 in the main text) were prepared, except containing each individual data point (for all CIEF linearity injections as opposed to means) to allow a statistical fit evaluation. The plots were fit to linear regressions of the form Y = m*X+b using the LINEST() function in Excel. Residuals were calculated based on the linear fit and residual standard deviation (rSD) and relative residual standard deviation (rrSD) were calculated for each fit. The rSD was calculated using Equation S5.

$$rSD = \frac{\sum (Y_{calc} - Y_{meas})^2}{n-2} \quad \text{(S5)}$$

where Y_{calc} is the theoretical Y value calculated from the line of best fit, Y_{meas} is the measured Y value, and n is the number of data points in the curve. The rrSD was calculated using Equation S6.

$$rrSD = \frac{rSD}{\sum Y_{meas}/n} \times 100\% \quad \text{(S6)}$$

Limit of Detection and Limit of Quantitation Calculations. Limit of Detection (LOD) and Limit of Quantitation (LOQ) were calculated as follows. The 6-sigmal signal-to-noise (SNR) ratio and relative abundance (RA) of the 1K basic variant were recorded in triplicate at the target loading concentration (0.4 mg/mL). The RA_{1K} was calculated as in equation S7.

$$RA_{1K}(\%) = \frac{CA_{1K}}{\sum CA_{all}} \times 100\% \quad \text{(S7)}$$

where CA is the time-corrected area determined by peak integration in 32 Karat. This peak was chosen because it exhibited SNR values in the range of 3-15 at the target loading concentration. The RA_{1K} was observed to be 7.0 (0.2) % (SD), and the SNR_{1K} was observed to be 13.9 (0.8). The LOD was calculated according to equation S8:

$$LOD(mg) = \frac{3}{SNR_{1K}} \times \frac{RA_{1K}(\%)}{100} \times C_{inj} \times V_{inj} \tag{S8}$$

where SNR_{1K} is the 6-sigma signal-to-noise ratio for the minor variant used for LOD calculation, taken from the 32Karat software, C_{inj} is the concentration of total protein loaded in the injection (0.4 mg/mL) and V_{inj} is the injection volume (5.89 x 10^{-4} mL). V_{inj} was calculated in CE Expert (v 1.0, Beckman Instruments, Brea, CA) according to Equation S9.

$$V_{inj} = \frac{\pi d^4 \Delta p}{128 \eta l} \times t \tag{S9}$$

where d is the capillary internal diameter in meters, Δp is the pressure drop across the capillary in Pascals, η is the buffer viscosity in Pascal-seconds, l is the total capillary length in meters, and t is the injection time in seconds. The LOQ was calculated as in equation S10.

$$LOQ(mg) = \frac{10}{SNR_x} \times \frac{RA_x(\%)}{100} \times C_{inj} \times V_{inj} \tag{S10}$$

The mass-based LOD and LOQ were converted to % relative abundance as in equations S11 and S12.

$$LOD(\%) = \frac{LOD}{C_{target} \times V_{inj}} \times 100 \tag{S11}$$

$$LOQ(\%) = \frac{LOQ}{C_{target} \times V_{inj}} \times 100 \tag{S12}$$

In the case of the CE assays discussed herein, $C_{inj} = C_{target}$ because a minor variant (1K peak for cIEF and 2K peak for CZE) was present at appropriate SNR for this type of determination.

Discussion of Thresholding in CIEF Data Analysis. The choice of threshold value in the 32 Karat data analysis software has a major impact on the type and quality of peaks that are included in the integration results. The software uses a default threshold level of 50, which must be optimized to be appropriate for a given method. The threshold level used for data analysis should be rationally chosen and be supported scientifically. The 32 Karat software package comes with a Graphical Programming tool which suggests appropriate integration parameters for a given electropherogram, including a suggested threshold level based on baseline noise. For the CIEF method developed in this work, thresholding is particularly crucial to the accuracy of the integration results because the background ampholytes are detected at 280 nm as integratable peaks in the baseline.

Figure S1 depicts the effect of various threshold levels on integration of ampholyte peaks in the baseline of a reagent blank injection. Integrated peaks are indicated by vertical ticks connected by a red horizontal baseline. A low threshold level of 100 results in most of the baseline peaks being included in the integration. Successive increases in threshold value result in fewer baseline peaks integrated. At the threshold value chosen for this method (1000), no baseline peaks are integrated.

The effect of these threshold values on integration of PS 8670 peaks is detailed in Figure S2. At a threshold of 100, both baseline and mAb peaks are integrated. Manual sifting of peaks is required to differentiate peaks of interest, which would introduce uncertainty between analysts with different judgements of what constitutes a mAb peak. At a threshold of 1000, a small amount of area attributable to the mAb is missed in the integration, but no manual data analysis

is required. Table S3 gives the relative abundances of the charge variants at the different threshold levels determined from triplicate sample preparations analyzed on one day by one analyst.

Table S3 PS 8670 Charge Variant RA at Different Threshold Levels Determined with cIEF

	**Mean (SD)[a]**	
	**[b]Threshold = 100**	**Threshold = 1000**
Main Group RA (%)	66.9 (0.4)	72.4 (0.4)
Acidic Group RA (%)	24.8 (0.5)	20.0 (0.5)
Basic Group RA (%)	8.3 (0.4)	7.6 (0.2)

[a]SD = Standard deviation. [b]Values corrected manually for peaks deemed to be ampholyte integration events.

Differences in measured charge heterogeneity values are due largely to differences in integration of the acidic group, reflecting poor separation efficiency and high background in this region. Major technology development beyond the scope of this work would be necessary to achieve high quality separation of the many charge variants comprising the acidic group. Barring this, we have erred on the side of conservative, automated integration in the interest of reproducibility and robustness over the lifetime of the product and method. The chosen integration parameters with a threshold level of 1000 are given in Table S13.

Capillary Zone Electrophoresis (CZE)

Preparation of CZE Background Electrolyte (BGE). The BGE employed for CZE analysis was the following, unless otherwise indicated: 400 mmol/L 6-aminocaproic acid (EACA)/2 mmol/L triethylenetetraamine (TETA)/acetic acid (pH 5.7), 0.03% (w/v) Tween^{TM} 20. The buffer was prepared as follows: 1) dissolved 26.234 g EACA and 149 µL TETA in ≈450 mL DIUF) water; 2) adjusted the pH to 5.7 using glacial acetic acid; 3) transferred the EACA/TETA/HAc solution to a 500 mL class A volumetric flask by passing through a 5.0 µm-pore PVDF syringe filter; 4) added 1.5 mL of 10% (w/v) Tween-20 (fresh); 5) brought the volume to 500 mL; and 6) mixed by inverting and shaking the flask 9 times. The final BGE was transferred to a tightly capped plastic storage bottle and stored at room temperature for up to 1 month. For buffers containing hydroxypropylmethylcellulose (HPMC), a 1% (w/v) solution of HPMC in DIUF water was prepared by dissolving 0.5 g HPMC in 50 mL DIUF water and mixing overnight to dissolve. EACA/TETA buffers were prepared separately from a 0.5 mol/L stock; HPMC was added at the indicated concentration immediately before use. HPMC-containing buffers were prepared fresh daily by mixing EACA/TETA and HPMC stock solutions.

CZE Method Linearity/LOD/LOQ Sample Preparation. In the case of linearity determination, samples were prepared (in triplicate) by serial dilution as in Table S4. The instrument qualification standard (IQ) was prepared by diluting 10 µL of the pI 10.0 peptide marker to 1/10X with 90 µL of DIUF water and mixing. Samples were prepared in Protein LoBind snap-cap tubes (Eppendorf, PN 89166-278 for 0.5 mL); then, 100 µL of each sample was transferred to a 0.2 mL CE sample vial for analysis (Sciex PN 144709 or Denville Scientific PN C18098-2).

Table S4 Serial Dilution of 8670 for CZE Linearity and LOD/LOQ Determination

**Concentration (mg/mL)**	**Volume of mAb**	**Volume of DIUF water**
2.5	80 µL of 10 mg/mL	240 µL
2.0	220 µL of 2.5 mg/mL	55 µL
1.5 (target)	175 µL 0f 2.0 mg/mL	58 µL
1.0	133 µL of 1.5 mg/mL	67 µL
0.5	100 µL of 1.0 mg/mL	100 µL
0.25	100 µL of 0.5 mg/mL	100 µL
0.025	10 µL of 0.25 mg/mL	90 µL

CZE Data Analysis. Electropherograms were analyzed using the 32Karat software package (Sciex Separations) using optimized integration parameters (Table S19 and S20). The integration parameters for the linearity/LOD/LOQ studies (Table S19) differ from the integration parameters used during qualification (Table S20) only in that the "Integration Off" command is set from 0 min to 8.5 min in the former and from 0 min to 4.0 min in the latter, to allow integration of the formulation buffer peak so that its retention time could be easily monitored. Retention times and corrected peak areas were recorded as in Table S5.

Table S5 Measured and Calculated Assay Results for CZE Electropherograms

Sample	Measurand Recorded	Calculated Parameters
Blank	Presence of new peaks (Y/N)	n/a
IQ	pI 10 Peptide migration time (min)	n/a
8670	Main peak migration time (min)<br>Main peak group corrected area<br>Basic peaks (2K peak, 1K peak) corrected areas<br>Acidic peak group corrected area	Main Group RA (%)<br>Basic Variants RA (%)<br>Acidic Variants RA (%)

Main group RA, basic variants RA, and acidic variants RA were calculated using equations S1, S2, and S3 above.

Statistics for CZE including intermediate precision were calculated in Microsoft Excel using the Analyse-it® plug-in (Analyse-it Software, Ltd., Leeds, UK) as discussed in [1]. Briefly, the precision for a given quality parameter was calculated by performing an ANOVA to estimate the total variance of the dataset and to model the components of the variance due to within-day variability (repeatability) and between-day variability (encompassing multiple columns, instrument drift, etc). This analysis was accomplished using the Analyse-it® measurement system analysis (MSA) Precision tool and setting the model to “Y with 1 random factor”, where the factor was the date of analysis. The estimator was set to be standard deviation with a two-sided 95 % confidence interval. The method was chosen to be “Exact/MLS”, and the “ANOVA” option was checked.

CZE Linearity Regressions. Plots of corrected area versus loading concentration (similar to Figure 10 in the main text) were prepared, except containing each individual data point (as opposed to averages) to allow a statistical fit evaluation. The plots were fit to linear regressions and statistical evaluations performed as described above for cIEF. The linear regression results for CZE data are described in Table S6.

Table S6 Features of Linear Regression Analysis of CZE Corrected Area vs Concentration Curves

**Plot**	**R-Squared**	**rSD[a]**	**rrSD[b]**	**F Statistic[c]**
Main Group	0.999	433.6	1.8 %	23202.228
Basic Group	0.998	94.3	3.3 %	7232.716
Acidic Group	0.996	239.5	4.4 %	3767.637

[a]rSD = residual standard deviation; [b]rrSD = relative residual standard deviation; [c]$F_{critical}$ = 4.667 corresponding to an alpha = 0.05.

CZE Limit of Detection and Limit of Quantification. LOD (mg), LOQ (mg) and LOD/LOQ (%) were calculated as discussed above for CIEF with the following exceptions. LOD/LOQ for CZE were calculated using the RA (%) and 6-sigmal SNR value for the 2K basic variant at the target loading concentration. This peak was chosen because it exhibited SNR values between 3-15 at the target concentration. The RA_{2K} was 0.59 (0.04) % (SD), and the SNR_{2K} was 8.9 (2.8) (n=3). C_{inj} was 1.5 mg/mL (C_{inj} = C_{target} in this case), and V_{inj} was 1.41 x 10^{-5} mL for CZE.

CZE Specificity. Method specificity was evaluated as discussed in the main text.

CZE Intermediate Precision. For determination of intermediate precision, samples were prepared daily from a fresh vial of PS 8670 as follows: thawed from -80 °C to room temperature, inverted 5 times to mix, centrifuged briefly, and diluted to 1.5 mg/mL with DIUF water. Dilutions of PS 8670 were prepared in triplicate on each day by mixing 15 µL of 10 mg/mL PS 8670 with 85 µL of DIUF water. On each day a blank sample was prepared by diluting 15 µL of formulation buffer with 85 µL of DIUF water. The instrument qualification (IQ) sample was prepared each day by mixing 10 µL of pI 10.0 marker peptide with 90 µL of DIUF water. Triplicate injection of each PS 8670 dilution (9 injections per day) was performed on three

different capillaries on six different days as outlined in Table S7. A fresh preparation of BGE was used with each capillary. The injection sequence for each day followed the form: Blank─IQ─8670 Prep #1 x3─IQ─8670 Prep #2 x3─IQ─8670 Prep #3 x3─IQ─Blank. Quality parameters were calculated and subjected to statistical analysis as described above.

Table S7 CZE Qualification Injections

Capillary	Day	Number of PS 8670 Injections
Capillary # 1	1	9
	2	9
Capillary # 2	3	9
	4	9
Capillary # 3	5	9
	6	9

Summary of Qualified CZE Method

CZE Method Performance Criteria. The IQ standard and PS 8670 will be used to evaluate system performance and system suitability, respectively, during NISTmAb RM 8671 value assignment [2]. Performance criteria for the method were set for each parameter based on the measured intermediate precision. These criteria are useful for ensuring that the analytical method is in control, thus establishing confidence in the data acquired using the method. The criteria for the IQ are as follows:

- Visually conforms to expectation (expected peak shape and pattern).
- The MT of the pI 10.0 peptide falls within $\pm 3u_c$ of the mean: (5.62 min to 5.96 min).

The criteria for injections of PS 8670 are as follows:

- Visually conforms to expectation (expected peak shape, no new peaks above LOD).

- The migration time of the main peak falls within $\pm 3u_c$ of the mean: (9.16 min to 10.19 min).
- The Main group RA (%) falls within $\pm 3u_c$ of the mean: (73.68 % to 75.71 %).

Blank injections should be performed at the start and end of each sequence and should contain no new peaks above the LOD.

Figures

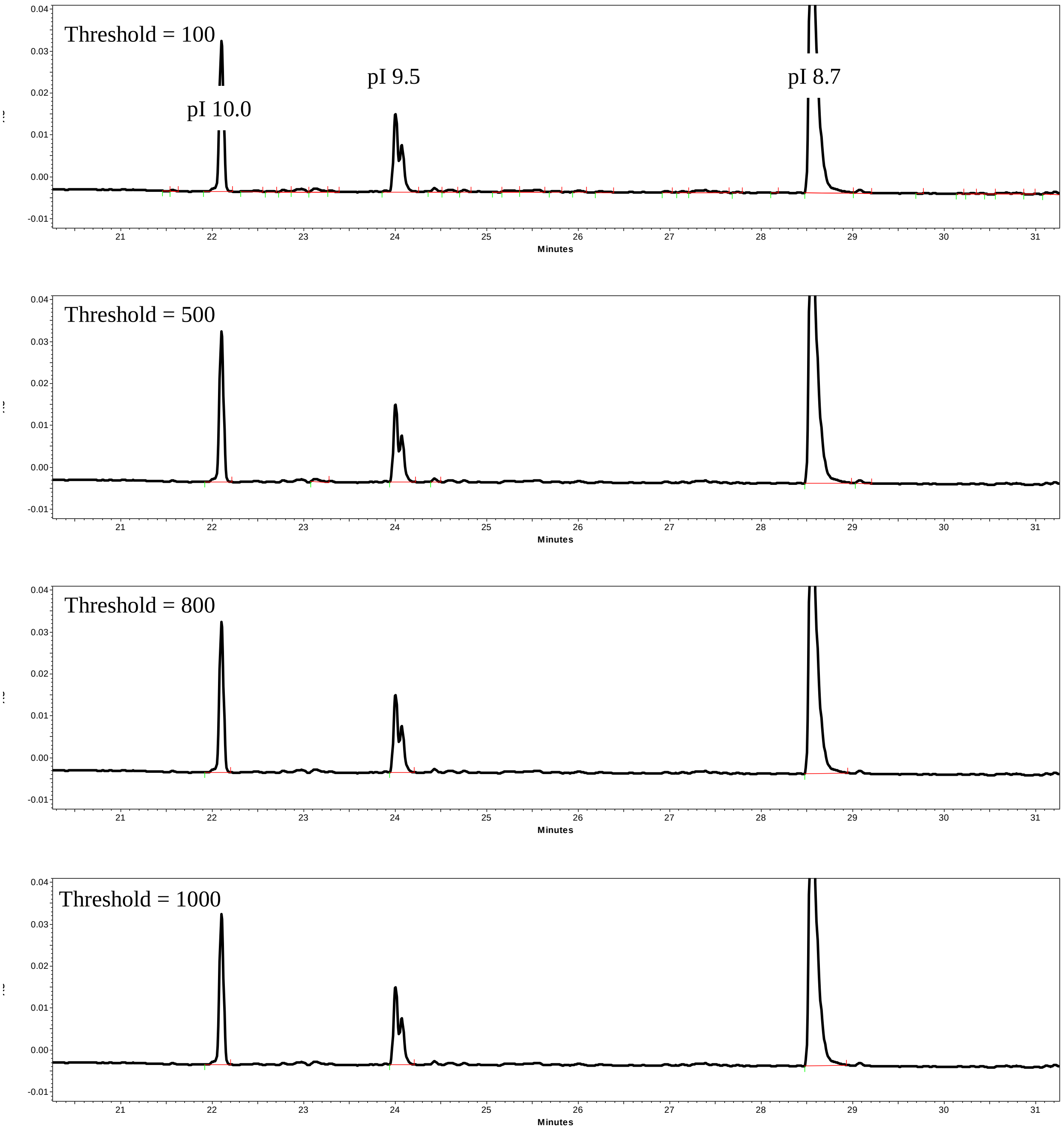

Fig. S1 Effect of 32 Karat Threshold Value on CIEF Integration of Blank Sample

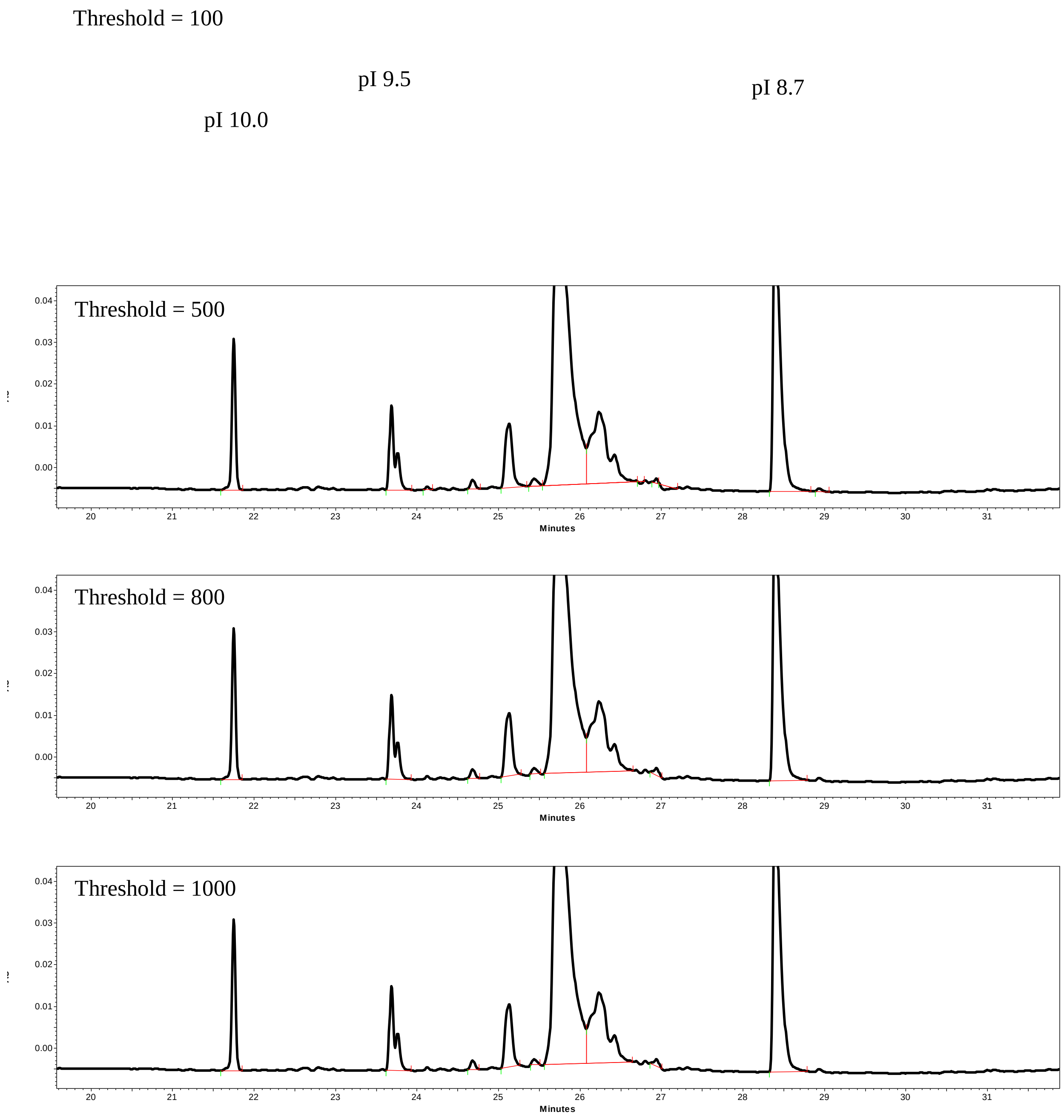

Fig. S2 Effect of 32 Karat Threshold Value on CIEF Integration of PS 8670

CIEF Instrument Methods

Table S8 Initial Conditions for CIEF Instrument Method

Parameter	Setting
Auxillary data channels	Current; max = 20.0 µA
Cartridge temperature	20.0 °C
Sample storage	10.0 °C
Peak detect threshold	2
Peak detect peak width	9
Trigger settings	Wait until cartridge coolant temperature is reached<br>Wait until sample storage temperature is reached
Analog output scaling factor	1
Inlet trays	Buffer: 36 vials<br>Sample 48 vials
Outlet trays	Buffer: 36 vials<br>Sample: No tray

Table S9 Initial Conditions for UV Detector in CIEF Method

Parameter	Setting
Electropherogram channel data	Acquisition enabled<br>Wavelength 280 nm<br>Data rate 2 Hz
Filter	Normal<br>Peak width: 16-25 pts
Absorbance signal	Direct

Table S10 CIEF Conditioning Method for New Capillaries: Time Program

#	Time (min)	Event	Value	Duration	Inlet Vial	Outlet Vial	Summary	Comments
1		Rinse-Pressure	50.0 psi	5.00 min	BI:F1	BO:B1	Forward	Chemical mobilizer rinse
2		Rinse-Pressure	50.0 psi	2.00 min	B1:B1	BO:B1	Forward	ddH20 rinse
3		Rinse-Pressure	50.0 psi	5.00 min	B1:E1	BO:B1	Forward	Urea-gel conditioning rinse
4		Wait		0.00 min	B1:A1	BO:A1		Idle position

Table S11 CIEF Separation Method Time Program

#	Time (min)	Event	Value	Duration	Inlet Vial	Outlet Vial	Summary	Comments
1		Rinse-Pressure	50.0 psi	3.00 min	BI:D1	BO:B1	Forward, In/Out vial inc 12	4.3 mol/L urea rinse
2		Rinse-Pressure	50.0 psi	2.00 min	B1:B1	BO:B1	Forward, In/Out vial inc 12	ddH20 rinse
3		Inject-Pressure	25.0 psi	99.9 sec	SI:A1	BO:B1	Override, forward	Sample injection
4		Wait		0.00 min	B1:A1	BO:A1	In/Out vial inc 12	Water dip
5	0.00	Separate-Voltage	25.0 KV	15.00 min	BI:C1	BO:C1	0.17 Min ramp, normal polarity, In/Out vial inc 12	Focusing step (urea-gel)
6	15.00	Separate-Voltage	30.0 kV	30.00 min	BI:C1	BO:D1	0.17 Min ramp, normal polarity, In/Out vial inc 12	Chemical Mobilization Step
7	45.00	Rinse-Pressure	50.0 psi	2.00 min	BI:B1	BO:B1	Forward, In/Out vial inc 12	ddH20 rinse
8	45.00	Stop data						Stop CIEF separation
9	47.00	Wait		0.00 min	BI:A1	BO:A1	In/Out vial inc 12	Water dip
10	47.10	End						Method end

Table S12 CIEF Shutdown Method Time Program

#	Time (min)	Event	Value	Duration	Inlet Vial	Outlet Vial	Summary	Comments
1		Rinse-Pressure	50.0 psi	2.00 min	BI:B1	BO:B1	Forward	ddH20 rinse
2		Rinse-Pressure	50.0 psi	5.00 min	BI:E1	BO:B1	Forward	Urea-gel rinse
3		Lamp-Off						Turn off lamp
4		Wait		0.00 min	B1:A1	BO:A1		Idle position

Table S13 Integration Parameters for CIEF Data Analysis

#	Event	Start Time	Stop Time	Value
1	Integration Off	0.000	15.000	0
2	Width	0.000	60.000	0.2
3	Threshold	0.000	0.000	1000
4	Shoulder sensitivity	0.000	0.000	9999

CZE Instrument Methods

Table S14 Initial Conditions for CZE Instrument Method

Parameter	Setting
Auxillary data channels	Current; max = 300.0 µA
Cartridge temperature	25.0 °C
Sample storage	15.0 °C
Peak detect threshold	2
Peak detect peak width	9
Analog output scaling factor	1
Inlet trays	Buffer: 36 vials<br>Sample 48 vials
Outlet trays	Buffer: 36 vials<br>Sample: No tray

Table S15 Initial Conditions for UV Detector in CZE Method

Parameter	Setting
Electropherogram channel data	Acquisition enabled<br>Wavelength 214 nm<br>Data rate 4 Hz
Filter	Normal<br>Peak width: 16-25 pts
Absorbance signal	Direct

Table S16 CZE Conditioning Method for New Capillaries: Time Program

#	Time (min)	Event	Value	Duration	Inlet Vial	Outlet Vial	Summary	Comments
1		Rinse-Pressure	50.0 psi	10.00 min	BI:D2	BO:D2	Forward, In/Out vial inc 9	Rinse with 0.1 N HCl
2		Rinse-Pressure	50.0 psi	20.00 min	BI:B2	BO:B2	Forward, In/Out vial inc 9	Rinse with BGE
3		Inject-Pressure	0.5 psi	10.0 sec	BI:C2	BO:C2	Override, forward	Inject buffer (dummy injection)
4	0.00	Separate-Voltage	30.0 KV	10.00 min	BI:C2	BO:C2	0.17 Min ramp, normal polarity, In/Out vial inc 9	Voltage equilibration for BGE
5	10.00	End						Method end

Table S17 CZE Separation Method Time Program

#	Time (min)	Event	Value	Duration	Inlet Vial	Outlet Vial	Summary	Comments
1		Rinse-Pressure	50.0 psi	3.00 min	BI:D2	BO:D2	Forward, In/Out vial inc 6	Rinse with 0.1 N HCl
2		Rinse-Pressure	50.0 psi	3.00 min	BI:B2	BO:B2	Forward, In/Out vial inc 6	Rinse with BGE
3		Inject-Pressure	0.5 psi	10.0 sec	SI:A1	BO:C2	Override, forward	Inject sample
4	0.00	Separate-Voltage	30.0 KV	15.00 min	BI:C2	BO:C2	0.17 Min ramp, normal polarity, In/Out vial inc 6	Separation (BGE)
5	15.00	End						Method end

Table S18 CZE Shutdown Method Time Program

#	Time (min)	Event	Value	Duration	Inlet Vial	Outlet Vial	Summary	Comments
1	0.00	Separate-Pressure	50.0 psi	5.00 min	BI:F2	BO:F2	Forward	Rinse with 0.1 M H_3PO_4
2	5.00	Wait		0.00 min	BI:A2	BO:A2		Dip capillary ends in ddH20
3	5.00	Lamp-Off						

Table S19 Integration Parameters for CZE Linearity/LOD/LOQ Data Analysis

#	Event	Start Time	Stop Time	Value
1	Integration Off	0.000	8.500	0
2	Width	0.000	0.000	0.2
3	Threshold	0.000	0.000	100
4	Shoulder Sensitivity	0.000	0.000	1e+006
5	Minimum Area	0.000	0.000	1000
6	Minimum Cluster Distance	0.000	15.000	5

Table S20 Integration Parameters for CZE Data Analysis During Method Qualification

#	Event	Start Time	Stop Time	Value
1	Integration Off	0.000	4.000	0
2	Width	0.000	0.000	0.2
3	Threshold	0.000	0.000	100
4	Shoulder Sensitivity	0.000	0.000	1e+006
5	Minimum Area	0.000	0.000	1000
6	Minimum Cluster Distance	0.000	15.000	5

References

1. Schiel JE, Turner A. The NISTmAb Reference Material 8671 lifecycle management and quality plan. Anal Bioanal Chem. 2018; https://dx.doi.org/10.1007/s00216-017-0844-2.
2. Schiel JE, Turner A, Mouchahoir T, Yandrofski K, Telikepalli S, King J et al. The NISTmAb Reference Material 8671 value assignment, homogeneity, and stability. Anal Bioanal Chem. 2018; https://dx.doi.org/10.1007/s00216-017-0800-1.
